# Providing effective and efficient hospital admission avoidance inpatient care: a systematic realist review of Norwegian municipal inpatient acute care services

**DOI:** 10.1186/s12913-026-14621-z

**Published:** 2026-04-29

**Authors:** Sujan Rijal, Marianne Sundlisæter Skinner, Simon Briscoe, Fan Yang, Rob Anderson

**Affiliations:** 1 Centre for Care Research, Department of Health Sciences in Gjøvik, Norwegian University of Science and Technology (NTNU), Helvin-bygget, C229, Teknologiveien 22, Gjøvik, 2815 Norway; 2 University of Exeter Medical School, University of Exeter, Exeter, UK

**Keywords:** Inpatient, Hospital admission avoidance intermediate care, Acute care, Quality of care, Efficiency, Cost, Utilisation, Patient satisfaction, Realist review, Realist synthesis

## Abstract

**Background:**

Healthcare spending in high-income countries has outpaced economic growth in recent decades, with hospital services accounting for a high proportion of it. Health systems in most high-income countries have explored models of intermediate care to provide less resource-intensive care outside hospitals. Different models of admission avoidance intermediate care have been introduced in countries with varying approach, capacity and settings e.g. hospital-at-home (HaH), inpatient admission avoidance intermediate care (AAIC) schemes. Although approaches like HaH have shown to be effective in providing care at a lower cost, inpatient AAIC schemes are less studied.

**Materials and methods:**

We conducted a realist review to capture evidence for explaining effectiveness in regards to utilisation and quality of care provided, and cost of admission avoidance intermediate care services. We searched for literature in three online databases (Ovid Medline, Embase and CINAHL) plus supplementary search methods (forward and backward citation searching) during October-December 2022 followed by a supplementary search in January 2026 to: (1) formulate initial programme theories and (2) refine programme theories. A total of 2625 studies from database search and eight more from manual searches were retrieved. After screening, full text of thirty five papers were explored out of which = analysis of eleven qualitative papers was included in the review for the formulation of programme theories which was further revised and refined through a stakeholder consultation. Seven empirical studies were used for refining the initial programme theories. From these, we developed seven programme theories and a theoretical framework, with researchers independently involved in theming data and developing explanatory causal chains of context-mechanism-outcome. Although the review was intended to seek evidence from inpatient admission avoidance intermediate care schemes from high-income European countries, most of the evidence originated from a particular scheme in Norway.

**Results:**

Three programme theories for explaining effectiveness related to referral to and utilisation of inpatient AAIC services: General practitioners’ (GPs’) positive/favourable perception of the care within inpatient AAICs; GPs’ confidence, security, and fulfilment of responsibility; and synergy and respect between healthcare providers. Two programme theories were related to quality of care: comfort and convenience for patients and an enabling working environment for nurses. Two programme theories were related to the cost of care: adaptive resources and payment mode. A causal framework for effective and efficient implementation and delivery of inpatient AAIC service was constructed.

**Conclusion:**

This review informs policymakers and managers towards successfully adapting inpatient AAIC services to specific settings. Intended outcomes are achieved when an enabling environment is ensured for both service providers and users. Flexibility in resource use facilitates efficient organisation of services. More primary research with a focus on the identification and evidencing of mechanisms - of how and why inpatient AAIC services work - is necessary to further inform the design and delivery of such services in different contexts.

**Supplementary Information:**

The online version contains supplementary material available at 10.1186/s12913-026-14621-z.

## Background

Healthcare spending in Organisation for Economic Co-operation and Development (OECD) countries has outpaced their economic growth in recent decades [1] raising concern about the financial sustainability of such spending Jakovljevic, Fernandes [2]. Expenditure in specialist health services accounts for a significant proportion of health system funding across OECD countries [3, 4]. A significant contributor to the pressure on specialised health services is the rise in inpatient admissions, where many individuals have been evaluated and deemed not in need of immediate or specialised care [5]. Thus, health systems in many high-income countries have experimented with models of intermediate care: hospital-at-home, early supported discharge, shared care- and other forms of ‘in-between care’ so that patients can receive less resource-intensive, appropriate and holistic care closer to home [6, 7].

Intermediate care aims to make healthcare more integrated and at a lower cost in the community compared to an acute hospital [8, 9]. Pearson et al., through their realist review concluded that improved service user outcomes are achieved in intermediate care due to increased user participation in decision-making, empowering service users for self-help, and increasing collaboration between different care providers [8]. Shepperd, Doll [10] describe admission avoidance intermediate care (AAIC) as a service that provides active treatment for conditions that do not require specialised healthcare services and can be treated either at home by healthcare professionals or at less specialised facilities. Without such services, these patients would be admitted to an acute hospital ward. Several countries have mostly adopted admission avoidance hospital at home to reduce unnecessary hospital admissions [10, 11]. Hospital-at-home potentially reduces costs to health service compared with hospital admission without any difference in patient’s self-reported health status [12]. Norway, Sweden and Denmark, however, have adopted inpatient AAIC services as an alternative [13], or in addition to at-home services [14]. However, with limited information available on schemes that are exclusively inpatient admission avoidance, there is a need to understand how and why such schemes work in different circumstances.

This review set out to capture and synthesise evidence from inpatient AAIC services from any high-income European country. However, initial enquiry of literature followed by application of our methods and predefined inclusion criteria meant that almost all relevant evidence originated from Norway. Thus, the review primarily comprises a review of studies dealing with the Norwegian model of inpatient AAIC model, i.e., Municipal Inpatient Acute Care (MIPAC) services, also labelled municipal acute units or wards (MAU or MAW) in the literature. We have for that reason, described the Norwegian AAIC service context in more detail below.

### The Norwegian context

The Norwegian health system is publicly financed through a tax system and has a semi-decentralized system with Regional Health Authorities (RHAs) responsible for specialist care and local government municipalities organising primary and social care services [15]. Specialist care is provided through 20 state-owned and governed public hospitals through block grants and case-based financing from the central government. Primary care and long-term care is provided at the municipal level by different providers: GPs (who act as gatekeepers to specialist services), as well as nurses and other medical/non-medical workers in municipal health and care services (for example nursing homes and home-based care) and these are financed by municipal taxes, block grants from the central government, National Insurance Scheme and patient co-payments [16]. However, universal health insurance does not totally cover long-term care but is instead funded through municipalities (i.e. local taxation) and patient copayments.

In 2012, as a result of health service coordination reform, Municipal Inpatient Acute Care (MIPAC) was introduced in all Norwegian municipalities with the aim of providing quality acute inpatient care closer to where people live at a lower cost compared to hospitals thereby reducing unnecessary hospital admissions and subsequent financial burden on the health system [13]. Although municipalities were free to organise services based on capacity, need and other local conditions, patient eligibility was nationally mandated [17]. The number of MIPAC services across Norway was 239 in 2021, representing 356 municipalities with a total of 685 beds which has increased to 688 in 2022 [18]. MIPAC services had to be (a) an inpatient service for acute conditions, (b) managed by the municipalities, and (c) exclusively meant to avoid hospital admissions.

MIPAC was initially planned to treat patients with known diagnosis for example patients with worsening chronic diseases such as heart failure, asthma or diabetes, in need of medication adjustments or with more acute but resolved disorders such as lung, kidney stones, falls, concussions. It was extended to either (1) stable patients with a clear diagnosis where the main problem is acute illnesses that could be treated using general medicine or worsening of known chronic illness requiring treatment or (2) Stable patients with unclear diagnosis not perceived seriously ill but need observation and investigation [17]. The service was later extended to people with mental or psychological health problems and drug or substance abuse problems.

## Methods

### Realist review

The realist approach of assessing interventions assumes that a singular deterministic theory might not always explain or predict outcomes in varying contexts [19]. Realist review methods can provide a more explanatory and context-sensitive approach to explain the semi-predictable reoccurring behavioural patterns by critically inspecting the interaction between contexts, mechanisms and outcomes using evidence from existing literature [15, 20]. Intermediate care services (and inpatient AAIC services) are services embedded in the already complex health system involving healthcare providers at different levels. Similarly, there are varying approaches to delivering admission avoidance intermediate care services within countries. Thus, we chose realist review as the method for this study to produce a contextualised understanding of the mechanisms by which inpatient AAIC schemes produce different patterns of outcomes.

We have conducted and reported this review in five broad stages as listed below. This review was guided by the Realist And Meta-narrative Evidence Syntheses: Evolving Standards (RAMESES) for realist reviews and we report it accordingly [16]. The realist review protocol was registered in the International Prospective Register of Systematic Reviews (PROSPERO) register before commencing the review (*registration number: CRD42023408350*) [21].

#### Identifying the scope of the review

The first step was to determine the scope of the review through exploratory literature searching and mapping out key themes and concepts. This enabled the reviewers to gain a deeper understanding of the research problem and refine the research questions:

1. What causal mechanisms, presented in literature, underlie the effectiveness and costs of inpatient AAIC services?
2. What are the relevant contexts, presented in literature, that enable (or hinder) the identified causal mechanisms to realise the intended outcomes (cost and effectiveness)?

#### Formulating initial programme theories

Initial programme theories in a realist review generally results from an iterative process within the research team and meeting with key stakeholders, who consented to participation, where potential theories are suggested. Key stakeholders included healthcare providers working within MIPAC services in Norway, comprising nurses and physicians from MIPACs with different organisational and municipal contexts, a civil servant from the national health authorities, and a carer organisation representative. This was followed by iterative analysis of primary studies. Bibliographic database searches were designed and conducted in three different electronic databases: Ovid Medline, CINAHL, and Embase, combining both free-text terms (e.g., title and abstract) and controlled vocabulary (e.g., MeSH in MEDLINE). This was conducted by an experienced information specialist [22]. We used combination of search terms such as “admission avoidance intermediate care”, “primary care centre”, “municipal acute unit”, “municipal acute beds” for care models, and “effectiveness”, “cost-effectiveness”, “programme evaluation” for outcome evaluation to find relevant articles {see supplementary file Explanation of methodology}. A total of 2625 sources were identified from the searches. The inclusion criteria for developing initial programme theories were qualitative research papers, policy reports or opinion pieces published: from 2000 onwards, in Norwegian or English about inpatient hospital admission avoidance services {for full inclusion criteria, see supplementary file Review protocol}. Manual searches for grey literature were conducted in Google Scholar, which generated eight sources.

10% of the publications obtained were initially screened by three reviewers independently to ensure uniform understanding and application of the inclusion criteria. The remaining studies were then screened by their titles and abstracts by two reviewers. Out of 2625 articles, 2595 were rejected after title and abstract screening. These articles were mostly quantitative studies of intermediate care models that were not inpatient and exclusively admission avoidance. Full texts of the 35 papers were then studied more thoroughly. Finally, papers were categorised according to their assessed theoretical richness. The categorisation of literature into conceptually: rich, thick, and thin sources was in accordance with the approach adopted by Pearson et.al. [8]. We rejected 24 studies that were conceptually thin. Eleven papers (10 conceptually rich and 1 conceptually thick) were used to formulate initial programme theories which is presented in Table 1.

**Table 1:** Studies included in the formulation of initial programme theories

Authors	Data collection	Participants	Sample	## PTs
Johannessen and Steihaug [23]	Interviews	Forty healthcare providers (GPs and staff in Norwegian MIPACs, purchasing offices and home-based nursing services)	Two MIPACs in Eastern Norway	1, 2, 4, 6,7
Nystrøm, Lurås [24]	Interviews	Twenty-one GPs	A single county in southeastern Norway	1, 2
Johannessen and Steihaug [25]	Interviews	Twelve patients and 40 healthcare providers from MIPACs, purchaser offices, and home-based services	Two MIPACs in Eastern Norway	1, 3, 4, 5
Leonardsen, Del Busso [26]	Semi-structured interviews	Twenty-three general physicians	Five MIPACs in south-eastern Norway	1, 2, 3
Leonardsen, Del-Busso [27]	Semi-structured interviews	Twenty-seven patients who used MIPAC services	Five MIPACs in south-eastern Norway	2, 4
Skinner [28]	Phone interviews, a case study of one MIPAC	Fifteen informants/focal persons for MIPAC service	Fifteen municipalities in Norway	1, 4
Gjerstad, Nødland [29]	Interviews	Fifteen COPD patients, sixteen managers and physicians in MIPAC	A single MIPAC in western Norway	2, 4, 5
Vatnøy, Karlsen [30]	In-depth interviews	Eight nurses and two physicians working in MIPAC	Five MIPACs in south-eastern Norway	5
Johannessen, Tveiten [31]	Interviews and observation	Eleven healthcare professionals	A single MIPAC in eastern Norway	5
Landstad, Hole [32]	Interviews	Ten physicians working in MIPAC	Two small towns in mid-Norway and seven rural municipalities	5
Deloitte [33]	Survey, interview and case study	Personnel in the municipality	Survey of 338 municipalities and Interview with 15 municipalities	1
Leonardsen, Del Busso [34]	Semi-structured interview	General practitioners	Five different MIPACs in southeastern Norway	2

**Fig. 1:**
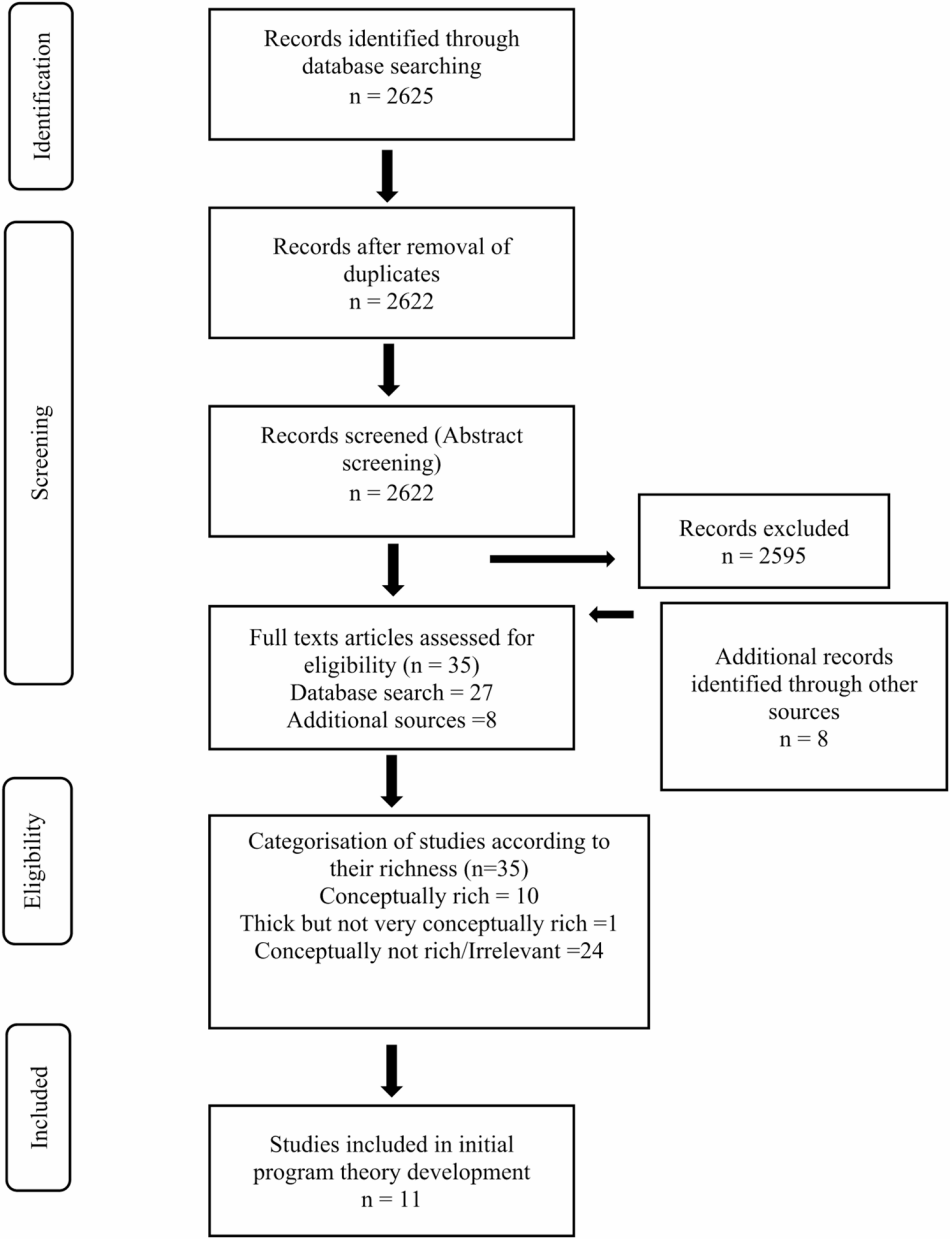
PRISMA diagram for stage 1 search

Initially, two researchers read through three articles independently to identify causal assertions or logical pathways. These were expressed as (If)contexts-(Then)outcome-(Because)mechanism statements (also referred to as programme theories from now on) and compared the results to improve the quality of relevant data extraction. This was followed by further data extraction and formulation of initial programme theories. Discussions were held between the researchers to translate information from the publications to insights about contexts and mechanisms. Consequently, emerging programme theories were recorded in a table and commented upon by the second reviewer (in MS Excel). Extensive consultation and review of the literature was conducted to ensure that all the relevant programme theories were captured. The flow of sources through the review and their use for identifying/formulating programme theories is shown in Fig. 1. {See supplementary file Formulated programme theories with sources for a complete list of initial programme theories}.

Nineteen AAIC specific initial programme theories were formulated and revised, that stemmed from MIPAC specific studies from Norway. They were sent to the study’s stakeholders, who assessed them based on their coherency, logical reasoning, or plausibility, and expected explanatory power. After incorporating feedback from the stakeholders, the 19 initial programme theories were combined into seven higher-level programme theories. The programme theories that operated through the same mechanism to produce the same outcome, albeit in different contexts, were combined into one more generic programme theory. The seven condensed programme theories were subjected to further refining, using evidence from empirical studies.

#### Searching for empirical evidence

The formulation of programme theories was followed by searches for evidence to find relevant body of empirical studies to further develop, refine and test the programme theories. This was mainly based on published findings from qualitative research about inpatient AAIC, process evaluations (e.g. mixed methods) and discussion sections of empirical evaluations. We systematically searched for primary empirical studies (qualitative and quantitative design) in three electronic databases: Ovid Medline, Embase and CINAHL (detail of search in supplementary file Explanation of methodology). Firstly, searches were run for literature that were specific to MIPAC in Norway which resulted in 2982 articles. Similarly, another round of search was carried out where interventions similar to MIPAC (in two aspects- exclusively admission avoidance and inpatient care) in European countries with dominantly taxation-funded healthcare systems were searched. This was done to maintain contextual comparability as including evaluation of schemes from countries where universal health coverage is provided through a mixture of public and private sectors. Named admission avoidance schemes were identified for some countries and listed for the search. This generated 371 studies in total. We applied filters: only papers from 2000 onwards and published in English for both the searches. Studies prior to 2000 would have been previously found and synthesised in an earlier realist review [8] intermediate care schemes prior to 2000 were dominantly for the purpose of both supporting early discharge and admission avoidance.

**Fig. 2:**
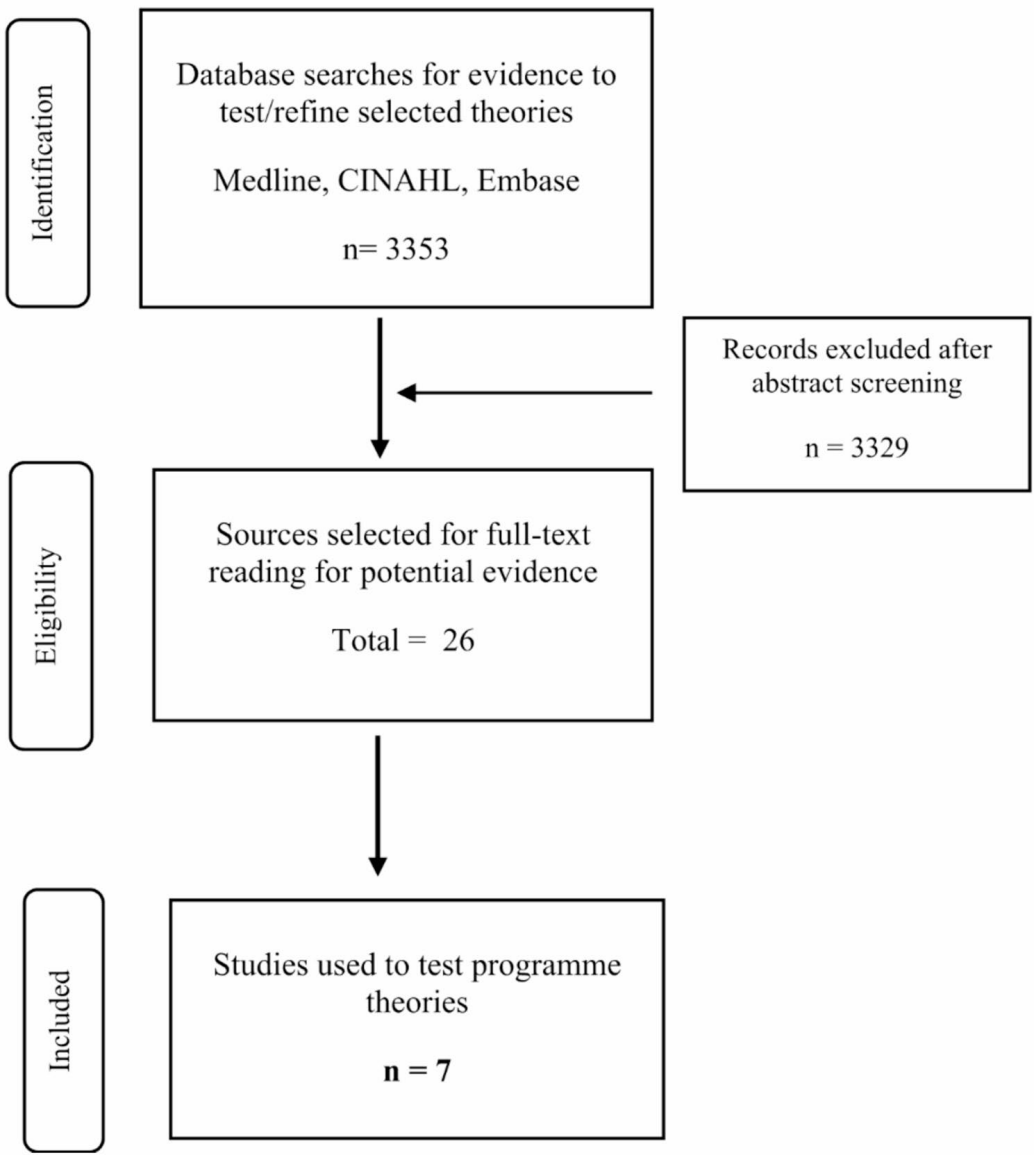
PRISMA diagram for search stage 2

Thus, a total of 3353 studies were selected for title and abstract screening. 3329 were rejected as they did not contain any evidence on the initial programme theories. We read full texts of 24 studies to assess if the studies included outcome evaluation (either efficiency or effectiveness) and some explanation of variations in outcomes. The process of searching and selecting literature for evidence has been shown in Fig. 2.

#### Appraising study quality and selecting relevant studies

Those studies that reported and/or provided explanation of variations in the outcomes of inpatient AAIC were included in this stage of the review. Six studies were found to include some form of evidence relating to the initial programme theories (five with evidence on Norwegian MIPACs and one from Australia). These six papers were subjected to critical appraisal using a tool developed by Wallace et al. broadly based on methodological soundness (e.g. clarity of research question and aim, study design, sampling, data collection and analysis) [35]. The qualitative studies used in formulation of initial programme theories were not included in this stage.

#### Extraction of data and refinement of theories

Data related to the setting of the study, objectives, methods, and results of the six studies identified to be relevant were extracted in MS-word. The papers were examined for: what effects (outcomes) did the introduction of AAIC scheme have? What caused these effects (identified or inferred mechanisms)? What were the underlying factors that the outcomes were realised (identified or inferred contexts)? Sections of all six papers identified to include evidence on specific initial programme theories (from one to seven) were extracted individually and reported. Key characteristics of the selected studies and evidence on specific programme theories can be found in supplementary file {Data Extraction}.

## Results

### Programme theories

The programme theories that relate to different contexts, mechanisms, and outcomes were refined through the extraction, analysis, and synthesis of evidence from the empirical studies. A brief excerpt of programme theories with corresponding studies used, along with description of each programme theory and related evidence obtained from the empirical studies has been presented in Table 2.

**Table 2:** Formulated programme theories with sources

PTs 1–3: Programme theories related to increased utilisation	Originating sources
**PT1: GPs’ positive/favourable perception towards AAIC**	
If referring GPs’ have a favourable attitude or perception towards and experience using inpatient AAIC services, they are more likely to refer patients to these services instead of hospitals. Contexts: Inter-municipal collaboration, geographical size of the municipality, proximity of inpatient AAIC service from municipality, physician availability, diagnostic facilities, use of on-call or substitute doctors, referral protocol/guidelines.	[23–26, 28, 33]
**PT2: Confidence in referring GPs in the services and their perception** **of having fulfilled their responsibility towards patients**	
If referring GPs’, (a) have confidence and trust in the medical competence of the service staff (doctors and nurses) and diagnostic facilities of the unit, (b) feel secure about patients’ safety, and (c) are satisfied about having fulfilled their responsibilities towards the patients, they will refer patients to the units rather than hospitals. Contexts: Out-of-hour physician availability in the units, nurses with advanced medical competence, diagnostic facilities, distance of inpatient AAIC units to hospitals, referral protocol/guideline	[23, 24, 26, 27, 29, 34]
**PT3: Synergy and respect between healthcare providers**	
If all the service providers understand and respect each other’s roles and work, they will collectively focus on patients’ needs and well-being. This, in turn, ensures timely referral and that appropriate care is provided. Contexts: Opportunities to interact with different service providers, knowledge/information sharing	[25, 26]
**PT4-5: Programme theories related to quality of care**	
**PT4. Comfort and convenience for patients** Organising and managing inpatient AAIC services that enable a sense of comfort and convenience^1^ for the patients results in high patient satisfaction, which assists in ensuring perceived quality of care. ^1^Comfort and convenience for patients refers to: easy accessibility, homely atmosphere, and frequent visits by relatives when the services are situated closer to patients’ residences, reduced waiting time and expedited care delivery compared to specialist health services, uncomplicated transfers, and increased privacy opportunities facilitated by the availability of private spaces. Contexts: Location of inpatient AAIC units closer to home, co-location of inpatient AAIC units with other health facilities, referral guideline/protocol, diagnostic facilities.	[23, 27–29]
**PT5. Enabling working environment for nurses**	
Although physicians are an indispensable part of care delivery, it is the nurses who are mainly responsible for most care delivery to the patients in the unit. If they feel safe, confident, and less stressed about care delivery and have the competency and adequate time for patient care, the care provided will be of high quality. Contexts: Education and experience of nurses, opportunity/time to focus on patient care, out-of-hour physician availability, diagnostic facilities.	[25, 29–32]
**PT 6–7. Programme theories related to cost of care**	
**PT6. Adaptable resources** If the resources such as infrastructure (building, beds, diagnostic facilities) and care staff (doctors, nurses, other staff) are flexible enough to be shared between different health facilities/institutions, this should assist the health facilities/institutions in achieving efficient delivery of care services through shared resource use. Contexts: co-location of inpatient AAIC units with other primary health care facilities like nursing homes or out-of-hours GP services.	[23]
**PT7. Payment model**	
Difference in the size of places/regions means there might be opportunities for neighbouring municipalities to cooperate in organising inpatient AAIC units with the larger municipality as host and smaller municipalities as collaborating partners. If the payment system is designed such that the partner regions located farther away from the host and with minimal demand for inpatient AAIC services must pay a fixed amount regardless of their utilisation of the services, it will be more expensive for the partners due to their lower demand for the services. On the contrary, if smaller municipalities have higher demand i.e. higher utilisation while paying the same amount, the effect is reversed. Contexts: Inter-municipal collaboration, geographical distance	[23]

Ensuring the flow of patients with defined care needs is a necessary pre-condition when implementing a health service innovation before the quality or cost of care becomes relevant. Thus, it is paramount that there are enough patients with need for inpatient acute care.

Firstly, for hospital admission avoidance inpatient care to be effective, patients must be referred to these service units and not hospitals. The first three programme theories are mechanisms relating to how GPs make decisions to refer patients to AAIC units. Secondly, the quality of care in these service units need to be good enough, if not better than hospitals, for the services to be appealing and be utilised as intended. The following two programme theories focus on ensuring quality in care delivery within the AAIC units. Lastly, for AAIC services to be efficient, they should build upon existing systems or infrastructure or resources and collaborate with health authorities from other areas. The last two deal with cost/efficiency of service delivery. A conceptual framework including mechanisms and outcomes for AAIC is presented as supplementary file {Conceptual framework for inpatient AAIC services}.

#### Increased utilisation

##### PT1: GPs’ positive/favourable perception towards AAIC increases referral

For the referring GPs to refer acute patients to AAIC services instead of hospitals, they need to have a favourable perception of the services. This depends on how informed they are or their first-hand experience of the units. GPs are more likely to refer patients to hospitals if they lack knowledge of the alternatives and the care they provide [36, 37]. The geographical size of the municipality and proximity of the AAIC services to the GP’s office affect their knowledge and perception. Smaller municipalities have fewer doctors to reach out to, and thus, the dissemination of information is less complicated compared to larger municipalities [36]. Health authorities in smaller regions or catchment areas have been found to be more successful in including all the GPs in planning the service compared to larger regions where only selected representatives are involved [36, 38]. Similarly, when two or more municipalities combine to establish joint AAIC services, the services are usually located far from the smaller partnering municipalities. This results in challenges to the dissemination of information. Moreover, in such a scenario, there is uncertainty about who is responsible for passing information about the offer, i.e. either the host municipality or the partnering municipality-ies. This challenge in information communication results in GPs not having a comprehensive overview of the service.

Strict and cumbersome referral and admission criteria/guidelines further exacerbate referral rates as GPs have to engage in negotiations with AAIC staff about the appropriateness of the patients. Furthermore, when GPs have to develop a treatment plan before referral, this leads to a time-consuming admission procedure at a pressing time due to the acute nature of the problems [23, 36, 37]. It has been reported that the admission process is more straightforward in AAIC services that are co-located with nursing homes, emergency departments or hospitals where there is availability of doctors during the day and night [36]. The opportunity to have communication with a physician in AAIC units, which ensures communication with someone possessing a similar level of professional knowledge promotes a positive attitude for the referring GPs [24]. Moreover, the use of on-call, substitute doctors in AAIC services means more complicated communication for the referring GPs compared to communicating with same physicians all the time.

##### PT2: Confidence in referring GPs in the services and their perception of having fulfilled their responsibility towards patients

GPs also need to have confidence and trust in the AAIC services and feel confident about their patients’ safety for them to refer their patients. One of the vital contexts is the presence of physicians in AAIC services, including out-of-hours. A majority of the patients are referred to AAIC services during the evening or nighttime, as seen in the Norwegian context [25]. If the units are staffed with physicians only in the daytime, referring GPs are worried about the safety of their patients [37] as they are not confident about the medical competence of the staff needed to provide appropriate care, and so refer the patients to hospitals instead [26]. Swanson and Hagen [27] found that there were statistically significant reductions in the admission rates to hospitals for municipalities hosting (-8.6%) and partnering with (-3.8% to -6.6%) AAIC service with physicians 24 h a day. Similarly, a study on the causal effect of the AAIC service on hospital admissions suggests that the presence of physicians on-site 24/7 in AAIC services reduced acute admissions to hospital medical departments by 3% for people over 80 years of age [28].

Most patients admitted to AAIC services are older and often have multiple conditions [25, 29]. Moreover, referring GPs have limited time considering the acute condition of the patient. Thus, it is important that AAIC services have basic diagnostic facilities, at least to facilitate prompt diagnosis. This strengthens the confidence of referring GPs and increases the referral of patients to the units [30, 31, 37]. GPs, thus, sometimes send patients to hospitals for preliminary diagnosis in municipalities where the AAIC services do not have diagnosis facilities and the geographical distance between facilities is not an obstacle [26, 37]. It has also been reported that the patients who are admitted to AAIC services after formal and comprehensive diagnosis have lower odds of being transferred to the hospital, which highlights the importance of the AAIC services having diagnostic facilities [25, 32]. Similarly, Hagen and Tjerbo [28] found that the presence of diagnostic opportunities in AAIC services, as a measure of ‘acute preparedness’, had an effect associated with a reduction in acute hospital admissions. This correlation suggests that the presence of diagnostic facilities is indicative of the readiness/preparedness of the services for receiving patients with acute conditions.

Crilly, Chaboyer [32] emphasise specific models of care delivery and roles undertaken by healthcare professionals during patient pathways are imperative to program utilisation. One key aspect highlighted is ensuring that each care provider has clear responsibility for patient care at any given time, minimising confusion. GPs have reported being sceptical about their responsibility towards the patient when referring to AAIC services. Unlike referring to hospitals, where the responsibility is transferred immediately to the hospital staff upon referral, GPs feel like they are still responsible throughout the patient journey when referring them to AAIC services. This leads them to choose hospitals over AAIC services Leonardsen, Del Busso [24].

##### PT3: Synergy and respect among service providers ensure appropriate referral to AAIC services

Numerous stakeholders are involved in delivering healthcare services, each with distinct aims, objectives, and responsibilities. They may interpret service function in a way that suits their responsibilities and workplace, which can disrupt a smooth patient pathway [32]. For instance, doctors and nurses in AAIC services prioritise delivering care to appropriate patients within their capacity and ability. They are wary of taking who might need specialised healthcare. In contrast, personnel from the health authorities may be more focused on maximising utilisation given that home-based services may be already at capacity and want patients to stay in the AAIC service as long as possible This contrast in objectives can occasionally create a conflict of interest, resulting in confusion and delays in healthcare decision-making Johannessen and Steihaug [23] exacerbated by gaps in communication [32]. Similarly, respect between AAIC staff and other (care and non-care) staff in the respective health authorities for each other’s work is vital for smooth coordination. Instances of disagreements regarding medical documentation in connection to patients have been reported [23]. It was argued that such collaboration across institutions was hindered by time pressures and a lack of opportunities to meet in-person.

#### Quality of care

##### PT4: Patient comfort/convenience and trust determine the perceived quality of care in AAIC services

Patient satisfaction is an important component of the quality of healthcare and a measure of how successful the healthcare system is in meeting expectations that are of most relevance to patients [33]. Patient perspectives have been positively associated with clinical effectiveness and patient safety of health services [34]. Services that are organised and managed to enable and foster patients’ sense of comfort and convenience often result in high patient satisfaction [39]. AAIC services with resources such that they have ample time to prepare for the patient by gathering vital information about the patient and preparing the room, explain the whole admission and treatment process, ensure patient involvement from the start, and provide continuous attentive care without haste were found to create trust and safety among the patients [30, 40]. There were also increased opportunities for personalised and informal communication, which patients appreciated [41]. Similarly, AAIC services with guidelines which enable flexible discharge, promote patient trust, as the care reflects the wishes of the patients to stay longer when there is a lack of immediately available long-term services [30, 36].

Patients also value a health service that is accessible – closer to home – which reduces the need to travel long distances to hospitals [40] and do not have to go through negative experiences in hospitals like overcrowded spaces and long waiting times [23]. Moreover, AAIC services located closer to home enable more frequent relative visits compared to hospitals, which patients appreciate [40]. Relatives often play a pivotal role in communicating patients’ conditions and needs and the level of treatment, especially given that those using AAIC services are often frail and in need of acute treatment and/or care. Thus, having AAIC services closer to home means that there is more opportunity for involving relatives to ensure a comprehensive assessment of the patient and appropriate treatment [37]. Availability of diagnostic facilities in AAIC services is perceived to be valuable by the patients. Firstly, having these facilities means they skip unnecessary visit to emergency wards of hospitals just to access those facilities before getting admitted back to AAIC services, and subsequent negative experiences in emergency wards of hospitals [37]. Secondly, they value being diagnosed at the earliest convenience and are sceptical about the quality of AAIC services when this is not possible [40].

##### PT5: Creating an enabling working environment for nurses ensures the quality of care in AAIC services

AAIC services rely on care mainly provided by nurses. Although physicians are vital in diagnosing, assessing patient conditions and devising treatment plans, nurses are responsible for providing continuous care. Thus, it is paramount that they feel confident and safe in delivering care and have the time and opportunity to do so. Nurses with broad medical knowledge, advanced clinical skills, ability to take a holistic approach to care, and previous experience working in acute care settings will be able to handle acute situations better [42, 43]. Vatnøy et al. suggest that the quality of care in AAICs depends on sufficient nursing staff in a professional and collaborating atmosphere acknowledged and supported by professional leadership [44]. Moreover, different skills workshops and on-site education increase the skills of nurses and may result in the avoidance of hospital admission [32].

Furthermore, having a physician available 24 − 7 and diagnostic facilities in the units often make nurses more confident about receiving patients and making decisions [23]. It was also noted that some nurses can feel so insecure about working alone without a physician during the night shift that they contemplate taking sick leave [23]. A 24 − 7 physician access also allows for a more precise division of responsibility, in some places resulting in the development of a transparent work plan and a flat and inclusive work environment, which is crucial for nurses to carry out their duties [30].

Lastly, nurses in AAIC units might be responsible for much administrative work, like documentation and day-to-day communication with health authorities. Some nurses have reported being overwhelmed by having to produce long reports, treatment plans, etc [43, 45]. This leads to nurses not having time to prepare units to receive patients or provide care to patients. Thus, a working environment with reduced administrative commitment which facilitates nurses to fulfil their essential role of providing care is a necessary mechanism to ensure that the services provided are of high quality.

#### Cost of care

##### PT6: Sharing resources with existing health institutions results in more efficient care delivery

AAIC services in municipalities with larger population generally have higher demand and utilisation, as data from Norwegian AAIC services suggest [46], which results in lower costs per patient or bed. Similarly, AAIC services can be and are often organised together with existing health institutions like nursing homes and GP out-of-hours services [26, 46]. Locating AAIC units with/in these existing health institutions means that the existing capital resources, like buildings and medical equipment, can be shared, which decreases the initial set-up cost. Similarly, the opportunity to utilise existing human healthcare resources (medical and non-medical) can help in the reduction of operation costs [26, 47]. Thus, it might be more efficient to establish and operate AAIC services within existing healthcare institutions.

##### PT7: Payment model determines cost-saving in inter-municipal collaboration

Different service catchment areas have varying demands for a particular health service and varying capacity to meet those needs. Municipalities that are restricted by their size and/or economy participate in inter-municipal collaboration to share the costs and risks of considerable investments to establish and provide AAIC services [48]. Thus, services might be organised in a way that the municipalities with larger population size which generally possesses readily available infrastructure and capacity, serve as the host organisation. The small(er) municipality(ies) are the collaborating partners, as seen in the Norwegian context [49, 50]. The partners have to compensate the host for the resource use. Usually, smaller municipalities experience fluctuations in service demand due to their demographic characteristics [51]. Moreover, the referral by GPs will be lower if AAIC services are located further away from where people live. Fan et al., reported MIPAC units with intermunicipal collaboration of more than five municipalities have lower chances of direct community admissions highlighting lower utilisation and use of MIPAC as step-down care [52]. Thus, an agreement where health authorities from such collaborating partners (geographically small and located further away) pay a fixed amount every year to the host, irrespective of service utilisation, can result in services being prohibitively expensive. This undermines the fundamental purpose of collaboration [26]. In contrast, the cost per patient for smaller municipalities with higher demand might result in cost-saving if they have fixed payment mode. They pay a fixed amount regardless of their higher use of the service. In this scenario, it could be more costly for the host municipality to deliver inpatient AAIC services. Thus, payment mode between municipalities is a vital mechanism in determining efficiency of care delivery.

## Discussion

### Summary of main findings

This review aimed to explain how different causal mechanisms and related contexts determine the effectiveness and efficiency of inpatient AAIC services. Seven initial programme theories (CMOs) generic to AAIC services were formulated which have been presented under three main themes after review of literature and stakeholder analysis.

A prerequisite for the AAIC service to be effective and efficient is enough demand for the service i.e. people who need AAIC services. All the programme theories described in the section below are under the assumption that there are enough patients who need AAIC services.

Firstly, we have identified mechanisms (and related contexts) related to patient referral and utilisation of services, which are primarily focused on the beliefs and decisions of referring GPs. For GPs to refer patients to AAICs they must have favourable attitudes towards, and confidence and a sense of patient security in the AAIC services. This is more likely to occur if they are provided with sufficient information about the AAIC services through involvement in all phases of program design and implementation such that they take some ownership of the service. Similarly, reducing the complexity of patient referral to the units and transparent responsibility for patients through clear referral guidelines, and increasing the medical capacity of AAIC services by extending physician availability throughout the whole day and establishing diagnostic services are all conducive to physicians referring more and more appropriately. AAIC services located “nearby” where people live are favoured by both GPs and patients due to shorter travel time. Similarly, AAIC services located very close to hospitals are less appealing to GPs and lead to underutilisation. It has been reported that chances of admission of patients to MIPAC increases as the distance of MIPAC to hospital ED increases [51]. Increasing opportunities for different healthcare providers in the primary healthcare sector to understand each other’s role in the health system better will enable them to respect each other’s work more. This ensures better coordination and appropriate patient referral.

Secondly, “quality of care” is an essential factor in assessing the effectiveness of AAIC services and is viewed through two perspectives: patient and clinical perspective. Patients appreciate it when AAIC services are located closer to home, which facilitates more visits and family/carer involvement in their treatment and rehabilitation. Similarly, services located in other primary care facilities means patients enjoy a more comforting “home-like” environment compared to hospitals, enhancing their overall experience. Moreover, shorter waiting times, adequate space, and opportunities for dialogue with staff owing to adequate time and capacity of the staff enrich the patient experience of the service. On the other hand, nurses who are at the core of ensuring the quality of care from the services need to have appropriate skills and experience working in acute care. They should also not be burdened with administrative work and allowed maximum time for patient care. Equipping services with 24 − 7 physician availability and diagnostic facilities will further improve patient care, and therefore also confidence in referring patients to the service.

Lastly, efficiency is an important issue while organising AAIC services. If AAIC services are organised along with existing healthcare services (wherever feasible) enabling flexibility in the use of resources, this can reduce fixed and/or operational expenses. Similarly, when two or more municipalities collaborate to establish AAIC services, the smaller municipalities utilise the medical expertise and infrastructure of the host municipalities (usually larger), reducing the financial risk. However, an agreement must be in place that ensures the partnering municipalities do not pay for unutilised services through a different payment mechanism e.g., a combination of capitation and fee-for-service. Thus, adopting a more flexible payment system that combines capitation and fee for service would perhaps be economically logical for the collaborating partners.

This is the first realist review of evaluation of services that are exclusively community-based (located closer to where people live) admission avoidance in-patient care. Pearson, Hunt [8] conducted a realist review of intermediate care in which literature on admission avoidance intermediate care schemes was included. While their findings relate to intermediate care more broadly, the programme theory from our review about patient satisfaction is consistent with their theories, i.e. improved service user outcomes are achieved when the place of care, and transition to it, are decided in consultation with the service user, and based on the location that is more likely to enable them to achieve their goals from the service. Similarly, the theory about synergy and respect among healthcare providers is consistent with Pearson et al.’s theory about the integrated approach of healthcare and service providers in that successful implementation requires active engagement and collaboration between different health and social care providers. They have also identified the importance of enabling the working environment in helping professionals to develop professionally and provide care to patients, which corresponds to our theory about creating an enabling working environment.

The first two programme theories are potentially specific to inpatient AAIC services in health systems where GPs act as gatekeepers. Increased utilisation in inpatient AAIC services is achieved when the referring GPs take ownership of the service and perceive it as medically safe when compared to hospitals. This is more important when inpatient AAIC services are introduced as new services within the health system, and the GPs have very limited or no information about the services. However, increasing the availability of doctors in inpatient AAIC services might be practically and economically not feasible. The programme theories about efficiency highlight an area that has been less studied. Although we acknowledge that collaboration between several municipalities for a health service might be distinctive to the Norwegian health system, we argue that these theories about efficiency in inter-municipal/area collaboration can have broader applications in other national health systems.

The programme theories do not explicitly address why countries like Norway have implemented inpatient AAIC services instead of strengthening home-based services. This might be partly explained by a perceived need for AAIC service due to an increase in the elderly population with high co-morbidities who need continuous medical attention and cannot be treated at home-based services. An approach where an acute care team is deployed to provide acute care services might be more effective in places with lower demand for AAIC services.

### Strengths/limitations of the review

This review was conducted and reported in line with RAMESES quality and publication standards. We used a realist synthesis framework to evaluate and explain inpatient admission avoidance intermediate care schemes, which, to the best of our knowledge, has not been done before. The findings of the review, although primarily generated from the health service developments and research of one specific country (Norway), are generic and related to stakeholders and processes that are similar across different health systems. They are generalisable to other similar, publicly funded health systems in high-income countries - perhaps especially those with hospitals managed by the central state and locally managed well-established primary care services.

Although a focus on ensuring favourable attitude and confidence in GPs, collaboration among service providers, and patient satisfaction as part of patient-perceived quality are not new, what this realist review achieves is to bring several kinds of research together to better explain effectiveness. Another strength is that the programme theories generated are from systematic and extensive database, and supplementary literature searches. The whole review process included multiple iterations and discussions among reviewers, consultation with stakeholders, ensuring consistent and credible development of programme theories based on empirical evidence. We also have identified CMOs for various stages of the AAIC scheme implementation process, which can be helpful for stakeholders trying to initiate new units or manage problems specific to a point along the implementation chain.

A key limitation of this review is the reliance on evidence primarily from Norway due to the absence of both AAIC initiatives and related research from other countries. This was mainly because community-based inpatient care services exclusively meant for admission avoidance are not as widely prevalent in health systems around Europe. There are initiatives that are in principle meant to avoid hospital admissions across Europe. However, they did not meet the inclusion criteria in certain aspects. For instance, most of the inpatient services meant to avoid hospital admissions were simultaneously used as step down care or early supported discharge care; in hindsight, we may have learned more if we had expanded our review scope to include these models of care from countries other than Norway. Since we were looking at services that are exclusively admission avoidance, most initiatives did not fit the criteria. Moreover, initiatives that are very similar to MIPAC embedded with different administrative or organisations frameworks, were often reported as grey literature or policy documents in the language of origin which was outside the scope of this study.

More research literature about AAIC initiatives in different settings/countries might have provided new programme theories about the intervention or given new insights into the identified mechanisms and contexts. Eleven studies were included in the formulation of the initial programme theories and six empirical studies in the more detailed refinement of programme theories, which is not ideal. Although we found multiple studies that studied the effects of the presence of contexts in the achievement of particular outcomes, there was limited evidence to inform the listed mechanisms (apart from a previous realist review). Thus, refinement of programme theories was conducted at a higher level of abstraction/generality to overcome this issue. Similarly, a realist approach is subjective and interpretive, especially in the context of a review of studies that are mainly qualitative research. Thus, different reviewers might have yielded a different set of CMOs. However, given the recurrence of similar ideas, participant experiences and causal assertions across a range of sources, we believe the main programme theories would likely emerge from reviews with the same aims.

### Recommendations for further research

The process and findings of this realist review enable us to make a few recommendations. The lack of empirical evidence to refine/refute our programme theories, (i.e. lack of empirical studies about mechanisms that drive the effectiveness of AAIC services) highlights the need for more explicitly theory-driven evaluations of AAIC services and provide an adequate description (qualitative and quantitative) of the critical contexts and mechanisms. This will help in gaining a better understanding of how AAIC services can be more effective, or the places or patient types where their implementation might need to be adapted. Similarly, considerable effort has been made to understand the effectiveness of such services, more primary evaluation studies on how AAIC services are deemed (or not) to be efficient are necessary to generate mechanisms related to cost and efficiency.

## Conclusion

The introduction of a new model of healthcare across a country requires a deeper understanding of the settings, contexts and mechanisms driving the success or failure of the new model of healthcare. Based on our findings, we suggest a set of mechanisms that policymakers in tax-funded health systems should think about while designing and implementing AAIC services. GPs are central to the utilisation of AAIC services, and so it is beneficial to create a sense of affinity, confidence, and ownership in them towards the service. This could be extended to including them in co-designing their local implementation, including their potential locations. Creating enabling environments for both patients and nurses helps in raising the overall quality of care in AAIC services. Finally, organising AAIC services within existing healthcare services and/or in collaboration with health authorities of other municipalities through dynamic payment partnerships might increase efficiency.

## Supplementary Information

Below is the link to the electronic supplementary material.

Supplementary Material 1: Data extraction and synthesis- detailed description of data extraction and synthesis

Supplementary Material 2: Explanation of methodology-Detailed systematic strategy used to search for articles

Supplementary Material 3: Initial programme theories- All stages in formulation of initial programme theories

Supplementary Material 4: Review protocol- protocol submitted to PROSPERO before conducting the review

## Data Availability

All the materials used for the review are attached as supplementary materials.

## Abbreviations

AAIC: Admission avoidance intermediate care
CMO: Context-Mechanism-Outcome
GP: General practitioner
HaH: Hospital at home
MAB: Municipal Acute Beds
MAU: Municipal Acute Units
MAW: Municipal Acute Wards
MIPAC: Municipal inpatient acute care unit
OECD: Organisation for Economic Co-operation and Development
PROSPERO: International Prospective Register of Systematic Reviews
RAMESES: Realist And Meta-narrative Evidence Syntheses: Evolving Standards
