# Supplementary material for: Providing effective and efficient hospital admission avoidance inpatient care: a systematic realist review of Norwegian municipal inpatient acute care services
Source: BMC Health Serv Res. 2026 Apr 29;26:834. doi: 10.1186/s12913-026-14621-z (PMC13270756; doi:10.1186/s12913-026-14621-z)
Supplement: Supplementary file 1 — Supplementary Material 1: Data extraction and synthesis- detailed description of data extraction and synthesis [file 12913_2026_14621_MOESM1_ESM.docx]

**Data extraction and synthesis**

Evidence to test programme theories was extracted to data extraction tables in excel. The evaluation of each source involved a critical assessment utilizing the Wallace et al. (2004) tool, as outlined in tables below.

1. **Author (Year): Vivian Nystrøm et al. (2022) #31**

| 1 | Question Is the research question clear? | Yes, the research question or research objective is clear. 1) describe the characteristics of patients intended as candidate for MAWs by primary care physicians, 2) explore the need for extended diagnostics prior to admission in MAWs, and 3) explore factors associated with patients being transferred from the MAWs to hospital. |
| --- | --- | --- |
| 2 | Study design Is the study design appropriate to answer the question? | Yes, the study design is appropriate. It has prospective, observational design, based on anonymous data collected. |
| 3 | Context Is the context or setting adequately described? | Yes, provided in table 1, page 2, col 2, bottom |
| 4 | Sampling Is the sample adequate to explore the range of subjects and settings? Has it been drawn from an appropriate population? | The samples are purposively chosen and of MIPACs chosen represents five MIPACs for 12 municipalities in South-eastern Norway. So, is not reflective of all the MIPACs in Norway |
| 5 | Data collection Was the data collection adequately described? Was it rigorously conducted to ensure confidence in the findings? | Yes, data collection has been adequately described mostly in page 3. Data was collected from 2014 to 2020 by nurses in the administrative positions at the MIPACs at the time of discharge. Individual level data were collected and thus can assumed to be valid and reliable. |
| 6 | Data analysis Was there evidence that the data analysis was rigorously conducted to ensure confidence in the findings? | Yes, descriptive statistics and logistic regression method was used for data analysis |
| 7 | Reflexivity Are the findings substantiated by the data and has consideration been given to any limitations of the methods or data that may have affected the results? | Yes, limitations to the data and methods used have been mentioned in the strengths and limitations of the study. *Page 10, col. 2* |
| 8 | Generalisability Do any claims to generalisability follow logically and theoretically from the data? | The authors have mentioned about the external validity of the data in the first paragraph of strengths and limitations. It follows the data collected and the samples used. |
| 9 | Ethics Have ethical issues been addressed and confidentiality respected? | The study was approved by REC and thus complies with all the ethical obligation. |

| **A** | **Source** |  |
| --- | --- | --- |
|  | Strengths and weaknesses | Based on a large and complete individual data set covering five MIPACs over a seven-year period. MIPACs of varying size, geographical location, staffing, diagnostic opportunities, etc. However, the analysis was more descriptive and explorative. They also mention about probable bias in selection of healthier patients for analysis. They also do not mention the characteristics of municipalities for each MIPAC although they listed the characteristics of each MIPAC |
|  | Source type | Quantitative research based on register data |
|  | Aim | 1) describe the characteristics of patients intended as candidates for MAWs by primary care physicians, 2) explore the need for extended diagnostics prior to admission in MAWs, and 3) explore factors associated with patients being transferred from the MAWs to hospital |
| **B** | **Topic** |  |
|  | Category | Patients admitted to MIPAC |
|  | Location(s) | 5 MIPACs in 12 municipalities in South-eastern Norway  MIPAC1: 11 beds, 2: 8 beds, 3: 10 beds, 4:4 beds, 5: 7 beds |
|  | Description of IC type | Hospital admission avoidance acute in-patient intermediate care |
| **C** | **Research methods** |  |
|  | Data sample and collection | 5 MIPACs working in intermunicipal collaboration for 12 municipalities. Data from all the patient aged 18 and above who were admitted in these MIPAC units from 2014 to 2022 were recorded before they were discharged like age, gender, treatment provided, main diagnosis, etc. with the help of a reporting form. |
|  | Participants | Patients aged 18 years and above admitted to MIPAC (n = 16 786). |
|  | Analysis | Descriptive statistics of variables recorded are presented. Univariate and multiploe logistic regression were conducted for one dependent variable at a time: patients needing extended diagnostics prior to MIPAC admission and patients being transferred from MIPAC to hospital. Outcomes were presented in the form of odds ratios and 95% confidence intervals. |
|  | Time of follow-up | Not applicable |
| **D** | **Evidence about programme theory#** | General findings- More patients were ‘admitted from an out-of-hours physician’ at the causality than from ‘a general practitioner’ (55.6% versus 41.1%).  Further, the MAW with the shortest ‘travel distance to the hospital ‘and ‘patients admitted at night’ had the highest odds of being needing extended diagnostics prior to MAW admission. |
|  |  | **PT2**- shows that 1 779 (10.6%) patients in the sample were assessed as needing extended diagnostics before admittance to a MAW. These patients were ‘older’, had more ‘comorbidities’ and had longer ‘length of stay’ compared to patients not needing extended diagnostics. P6, c2  **PT16**- Patient ‘being admitted to MAW after extended diagnostics’ had lower odds for being ‘transferred to hospital’. P8, c1  **PT14**- In the multiple regression model, the variables describing advanced medical treatment (i.e., ‘intravenous fluid  therapy’, ‘intravenous medications’, and ‘oxygen therapy’) had higher odds of transfer to hospital. P8, c1  **PT18**-Our finding show that the medical treatment provided at the MAW is rather basic and hence could alternatively have been managed at home with the help of home healthcare services. This indicates that the home healthcare services capacity or competence might be too low. Hence, capacity building in home healthcare services might further reduce the pressure on hospitals.  **PT3**- Moreover, our results show that patients admitted to the smallest MAW who also had the longest travel distance by car to the hospital were most likely to be transferred to hospital. In contrast, patients admitted to one of the biggest MAWs with shorter travel distance by care to hospital had lower odds for being transferred to the hospital. This may indicate that healthcare personnels are more uncomfortable managing the risks of treating acutely ill patients when they are further away from the hospital. P8,c2.  **PT-15** The extended diagnostics in hospital was used for one out of ten patients. The extended diagnostics service has become a better-known opportunity for clinicians to ensure that patients admitted to the MAW receive the right diagnosis; that is, the use of X-rays, laboratory tests and specialist assessment by hospital physicians are assumed to give the patients a more clarified diagnosis [7, 12]. However, most patients in MAWs are old, with a high degree of frailty [12, 19]. The risks of transporting frail elderly individuals are diverse, and their transport is associated with a significantly increased risk of morbidity and mortality [29–31]. Therefore, the decision to transport patients to hospital for extended diagnostics before admittance to MAW should be based on a weighing of the necessity of being diagnosed in hospital against the potential risk involved. A frailty index could help to identify older people at risk of health decline and mortality, guiding clinicians in their decision-making [32]. P9,c1  **PT11**- There has been a scepticism about the lack of physician coverage at MAWs throughout the day [12, 33]. It may be argued that for the MAW to be an acceptable alternative to hospitals, their equipment and expertise must be similar to those of hospitals. However, there are several studies that indicate that there is no threat to patient safety to be treated in nurse-led units [34, 35], and two single centre randomised controlled studies found reduced morbidity after treatment in this kind of decentralised healthcare service [36, 37]. In a prospective observational study, it was shown that a ‘triage early warning score (TEWS)” above 2 indicates that patients have critical symptoms, need advanced treatment, and are more likely to be transferred to hospital from a MAW [10]. Hence, implementing the use of the TEWS score at diagnosis may guide clinicians in deciding which patients are suitable for admission to a MAW and which patients should be admitted to hospital. P9, c1 and 2. |

1. **Author (Year): Vatnøy et al. (2020) #33**

| 1 | Question/objective Is the research/objective question clear? | Yes, the research objective is clear and was to get an overview of the nursing competence in MIPAC units across geographical regions, and different groups of organisation and localisation. |
| --- | --- | --- |
| 2 | Study design Is the study design appropriate to answer the question? | Yes, the study has a cross sectional design with descriptive statistics through ad hoc questionnaires sent to the first line leaders in all MIPAC units. |
| 3 | Context Is the context or setting adequately described? | Yes, the setting and contexts are adequately defined in pages 2 and 3 under background and setting section. |
| 4 | Sampling Is the sample adequate to explore the range of subjects and settings? Has it been drawn from an appropriate population? | Questionnaires were sent to first line leaders of 226 MIPACs in Norway out of which 207 (91.6%) responded. So, the sample is thus adequate. |
| 5 | Data collection Was the data collection adequately described? Was it rigorously conducted to ensure confidence in the findings? | Data was collected from 207 MIPACs with the help of a web-based questionnaire which was based on a previous qualitative study. Leaders from 19 MIPACs did not respond. |
| 6 | Data analysis Was there evidence that the data analysis was rigorously conducted to ensure confidence in the findings? | Yes, descriptive analysis followed by non-parametric statistical tests were carried out as the data were not normal. P-values were Bonferroni-corrected to avoid type-1 error and effect size was calculated for pairwise comparisons. |
| 7 | Reflexivity Are the findings substantiated by the data and has consideration been given to any limitations of the methods or data that may have affected the results? | Yes, limitations of cross-sectional design in describing changes over time has been mentioned. Authors mention the unavailability of some analyses due to heterogeneity of the data. They also mention the fact that nursing competence is a multi-faceted phenomenon and might be explored and explained differently. |
| 8 | Generalisability Do any claims to generalisability follow logically and theoretically from the data? | There are no specific claims to generalisability except the authors mention that they tried to provide a complete overview of all MIPAC locations which was harder to achieve and there might be some units which act as MIPAC that were missed. |
| 9 | Ethics Have ethical issues been addressed and confidentiality respected? | The study was approved by REC and thus complies with all the ethical obligation. |

| A | Source |  |
| --- | --- | --- |
|  | Strengths and weaknesses | Authors tried to collect data from all the MIPAC units instead of taking a small sample which is helpful in trying to explore variations in the nursing competence in MIPACs in Norway.  Cross-sectional design of the study meant limitations in describing changes over time has been mentioned. |
|  | Source type | Data from a web-based questionnaire from a cross sectional quantitative design |
|  | Aim | To get an overview of the nursing competence in MIPAC units across geographical regions, and different groups of organisation and localisation. |
| B | Topic |  |
|  | Category | MIPAC units |
|  | Location(s) | 209 MIPACs all around Norway |
|  | Description of IC type | Hospital admission avoidance acute in-patient intermediate care |
| C | Research methods |  |
|  | Data sample and collection | n=209 MIPACs from different geographical locations, co-locations, sizes, etc. were used as sample out of 226 MIPACs. First line leaders were sent a web-based questionnaire consisting of questions about location, intermunicipal collaboration, total number of beds, and details about nursing competence. |
|  | Participants | First line leaders from 209 MIPACs |
|  | Analysis | Descriptive analysis of numeric variables. Two independent two sided non-parametric tests i.e. Mann-Whitney U test and Kruskal-walls test to assess association between groups i.e. comparison of registered nurse (RN) ratios, shifts with one RN, nightshift with one RN and dayshifts with one RN for MIPAC groups based on institutional locations, geographical regions and organisational categories. |
|  | Time of follow-up | Not applicable |
| D | Evidence about programme theory# | The number of RNs at the MipACs holding master’s degree or specialised competence was in general low. The lowest ratio of RNs to other staff and the highest frequency of shifts with only one RN on duty was found in MipACs located at nursing homes, organised together with a long-term care unit, and in MipACs in the Northern and Central regions of Norway. P6, c2.  **PT11-** The many benefits of a high proportion of RNs working bedside in general is described in several studies. A high ratio of RNs to other staff have showed positive impact on patient safety  and patient satisfaction, but also on nurses’ job satisfaction and prevention of burnout as well as expensive and disruptive turnover of staff [5, 42]. Furthermore, RNs working in municipal health care services are found to focus more on the patients’ life situation in a holistic perspective compared to  those working in hospital emergency departments [53].  PT12- the overall nursing competence and quality of care depend on sufficient nursing staff in a professional and collaborating atmosphere acknowledged and supported by a professional leadership. Nurses holding advanced nursing competence is also shown to safeguard patients with  complex health care needs in rural areas, as they are found to deliver high adherence to clinical guidelines and provide diagnostic accuracy (24).  and provide diagnostic accuracy [24]  **PT4**- Although the lack of nursing competence revealed in our study does not necessarily provide evidence for compromised safety and quality in patient care in Norwegian MipACs, it might support the assumptions that low competence can partly explain the low use of MipAC beds in general [15, 16, 33]. |

1. **Author: Swanson et al. (2016) #39**

| 1 | Question/objective Is the research/objective question clear? | To investigate whether implementation of MIPAC resulting from the  Norwegian Coordination Reform (2012) was associated with reductions in hospital admissions, particularly for the elderly. |
| --- | --- | --- |
| 2 | Study design Is the study design appropriate to answer the question? | A municipality-based retrospective comparative cohort study using monthly population-based registry data analysed with fixed-effects log–log regressions |
| 3 | Context Is the context or setting adequately described? | Yes, the setting has been described which is Norwegian municipalities and hospitals but the not much has been said on the variations in the municipalities in terms of size, population, health services. |
| 4 | Sampling Is the sample adequate to explore the range of subjects and settings? Has it been drawn from an appropriate population? | All the patients getting service from Norwegian hospitals between 2010 and 2014 are the subjects of the study. No sampling needed. |
| 5 | Data collection Was the data collection adequately described? Was it rigorously conducted to ensure confidence in the findings? | Data on patients admitted to hospital in Norway between 2010 and 2014 excluding psychiatric hospitals was collected obtained from National Patient Registry, Norway. Municipal data were collected from the database KOSTRA and telephone interviews. |
| 6 | Data analysis Was there evidence that the data analysis was rigorously conducted to ensure confidence in the findings? | Yes, there is evidence that the data analysis was rigorously conducted and has been documented well under analysis section. |
| 7 | Reflexivity Are the findings substantiated by the data and has consideration been given to any limitations of the methods or data that may have affected the results? | The findings are substantiated by the data. |
| 8 | Generalisability Do any claims to generalisability follow logically and theoretically from the data? | They mention that the results might not be generalisable to municipalities that had not opted to introduce MIPAC yet. But they have considered various factors to make the analyses generalisable under the analysis section. |
| 9 | Ethics Have ethical issues been addressed and confidentiality respected? | Ethical approval granted by the Regional Ethics Committee South-East, Norway; and the Norwegian Data Inspectorate. |

| A | Source |  |
| --- | --- | --- |
|  | Strengths and weaknesses | The major strength of this study was the exact starting dates and implementation range of MAUs over the study period  making it uniquely possible to isolate the effects of MAUs on the number of hospital admissions.  An important limitation is the narrow time period after the reform’s implementation and lack of 50% of the municipalities yet to adopt MAU services. Another possible endogeneity concern occurs from municipalities expecting demand for hospital admissions to rise, introducing MAU services, and this introduction subsequently prevents noticing the rise in figures, ultimately biasing the estimates of the effect towards zero. |
|  | Source type | Retrospective observational study |
|  | Aim | to evaluate the use of a small MIPAC in rural area regarding occupancy rate, patient characteristics and healthcare provided during the first four years of operation |
| B | Topic |  |
|  | Category | Norwegian hospitals and municipalities |
|  | Location(s) | Norwegian hospitals and MIPACs (until 2014) |
|  | Description of IC type | Hospital admission avoidance acute in-patient intermediate care |
| C | Research methods |  |
|  | Data sample and collection | No sample needed as registry data from 2010 to 2014 was used. Data from all the functional MIPAC until 2014 was also collected. |
|  | Participants | All patients who were admitted to the Norwegian hospitals from 2010 to 2014. |
|  | Analysis | Descriptive statistics and regression analysis followed by sensitivity analyses was done. In regression, demand for hospital services from each municipality was the dependent variable with municipal supply such as nursing homes, municipal demand factors like age composition of the population, and location of MIPAC alone or with another health institution as the independent variables. Detailed explanation of analysis is presented in p3 and 4 of the paper. |
|  | Time of follow-up | NA |
| D | Evidence about programme theory# | **PT-4:** There was an overall effect of MAUs with a physician on duty 24 hours a day, 7 days a week, which was generally  far stronger than the general effects reported above. For municipalities operating MAUs as hosts providing physician  services 24 hours a day, 7 days a week, there were significant changes in admission rates of −8.1% (p<0.01) for acute admissions and −8.6% (p<0.01) for acute admissions at internal medicine departments. The remaining interaction effects characterising services and location for MAU hosts were nonsignificant, indicating that having physicians on duty is a  crucial factor. The effects were same for MAUs operating alone although the effect was slightly weaker (−1.7% (p=0.03) for acute admissions and −1.7% (p=0.02) for acute admissions at internal medicine departments).  For municipalities using MAUs as visitors, the effect ranged from −3.8% (p<0.01) to −6.6% (p=0.02) for acute admissions and from −4.0% (p<0.01) to −6.5% (p=0.02) for admissions to acute internal medicine departments depending on the availability of physician services and MAU location or combinations thereof. Also, for this group of municipalities, the results indicate that MAUs without either around-the-clock physician services or operating in conjunction with an local emergency service had non-significant effects.  Significant effects of MAUs can mainly be found in medium-to large municipalities. We speculate that demographic and  geographic characteristics of smaller, often more remote municipalities have led to the development and usage of  services and provisions substituting and complementing hospital care in these areas prior to the introduction of the MAU mandate—a factor not captured by the fixed effects nor the factors controlled for by the selection of independent variables |

1. **Author: Hagen et al. (2023) #40**

| 1 | Question/objective Is the research/objective question clear? | Yes, the research objective was the paper was to investigate whether the implementation of MAWs had a causal effect on hospital admission. |
| --- | --- | --- |
| 2 | Study design Is the study design appropriate to answer the question? | Yes, the study design is appropriate to answer the question. They have used panel data regression method to analyse the causal effect. This is a retrospective design using registry data from 2010 to 2017 which is appropriate for such analysis. |
| 3 | Context Is the context or setting adequately described? | The Norwegian context of the coordination reform 2012, introduction of MAWs in Norway as an alternate to hospitalisation have been well described. |
| 4 | Sampling Is the sample adequate to explore the range of subjects and settings? Has it been drawn from an appropriate population? | No sampling needed as panel data has been used. |
| 5 | Data collection Was the data collection adequately described? Was it rigorously conducted to ensure confidence in the findings? | The study population and data source have been adequately described under data and methods. |
| 6 | Data analysis Was there evidence that the data analysis was rigorously conducted to ensure confidence in the findings? | Yes, the data analysis was rigorously conducted. They have used fixed effects in data with panel structure and have been explicitly described under statistical model and analyses section of the study. |
| 7 | Reflexivity Are the findings substantiated by the data and has consideration been given to any limitations of the methods or data that may have affected the results? | The findings are based on national level data for 8 years which is substantial to find any causal effects. They have used data from municipalities along with patient level data from NPR. Their demand model was formalised within a generalised framework for policy analyses of panel data.  There are however some limitations mentioned in the article of the method. Endogeneity due to the self-selection by the municipalities to introduce MAWs is a caveat to the method. Similarly, lack of patient level data from MAWs was a bigger problem in the generalisation. |
| 8 | Generalisability Do any claims to generalisability follow logically and theoretically from the data? | The generalisability follows the findings from the data. |
| 9 | Ethics Have ethical issues been addressed and confidentiality respected? | As only aggregated data have been used, approval from ethical committees was not required. The study has undergone ordinary quality assurance at the University  of Oslo and is registered in the Forskpro-register as part of the NORCHER-project. |

| A | Source |  |
| --- | --- | --- |
|  | Strengths and weaknesses | **Strength-** The findings are based on national level data for 8 years which is substantial to find any causal effects. They have used data from municipalities along with patient level data from NPR. Their demand model was formalised within a generalised framework for policy analyses of panel data. The use of foxed effect also allows to control for heterogeneity and focus on the variable of interest.  **Weakness**-Endogeneity due to the self-selection by the municipalities to introduce MAWs is a caveat to the method. Similarly, lack of patient level data from MAWs was a bigger problem in the generalisation. |
|  | Source type | Quantitative study (using panel data from national registry) |
|  | Aim | to investigate whether the implementation of MAWs had a causal effect on hospital admission |
| B | Topic |  |
|  | Category | Norwegian hospitals and municipalities |
|  | Location(s) | Norwegian hospitals and MAWs (until 2017) |
|  | Description of IC type | Hospital admission avoidance acute in-patient intermediate care |
| C | Research methods |  |
|  | Data sample and collection | No sampling needed as patient level data from National Patient Registry on the use of hospital services from 2010 to 2017 was used. Similarly, municipal data from obtained from municipal registry KOSTRA and through telephone interviews. |
|  | Participants | All the patients who used hospital services from 2010 to 2017. |
|  | Analysis | Fixed effects in data with panel structure was used for analyses. All the variables except MAW was exogenous. Some endogeneity problems were handled through the municipal fixed effects. Descriptive statistics along with regression analyses were done and presented. The number of acute admissions for patients was used as the dependent variable. |
|  | Time of follow-up | NA |
| D | Evidence about programme theory# | **PT4**- For the oldest age group, the effects of the introduction of MAWs on acute admissions in medical departments was minus 2 percent. There was an additional effect of minus 1 percent if the MAW was organized with a physician on site 24/7 or was located close to a local emergency centre. For the age group <80 years, the reduction in acute admissions could be found only among municipalities that had a MAW with the highest level of acute preparedness.    **PT2 and PT4**- For patients <80 years of age, only the MAWs with the highest acute preparedness had an effect. This is important as it indicates that the level of acute services present in the intermediate unit is crucial. (Diagnostic opportunities, lower medical expertise throughout the day, uncertainty about the selection of patients and challenges with user participation). |

1. **Author: Hilland et al. (2023) #41**

| 1 | Question/objective Is the research/objective question clear? | The objective was to contribute to the discourse by studying how the introduction of intermediate care in the form of municipal acute units (MAUs) in Norway has affected age adjusted mortality rates and hospital readmissions. |
| --- | --- | --- |
| 2 | Study design Is the study design appropriate to answer the question? | Yes, the study design of retrospective cohort study using population-based registry data from 2010 to 2016 analysed with fixed effect regression. |
| 3 | Context Is the context or setting adequately described? | The context of introduction of MAUs has been well documented. MAUs have been defined well and the rationale behind the introduction have been mentioned well under introduction section. |
| 4 | Sampling Is the sample adequate to explore the range of subjects and settings? Has it been drawn from an appropriate population? | Population level data from national registry data from 2010 to 2016 have been used which means that there was no sampling was required. |
| 5 | Data collection Was the data collection adequately described? Was it rigorously conducted to ensure confidence in the findings? | Registry data was used as mentioned above which is patient level data from the national registry. |
| 6 | Data analysis Was there evidence that the data analysis was rigorously conducted to ensure confidence in the findings? | Yes, the data analysis was rigorously conducted with the regression model accounting for confounders such as geographical factors, structural factors, etc. |
| 7 | Reflexivity Are the findings substantiated by the data and has consideration been given to any limitations of the methods or data that may have affected the results? | Although the case for generalisation is strong with the use of panel data over 6 years, there is a limitation in terms of generalisations of the effects if MAUs in the smallest municipalities due to some missing data from those municipalities.  Some other limitations mentioned were: short time period under study, the use of municipal aggregated as opposed to individual registry data which means that some of the effects of MAU on the outcome might not explained with the variables in the study but broader changes to the healthcare system. |
| 8 | Generalisability Do any claims to generalisability follow logically and theoretically from the data? | Yes, the generalisability follow logically from the data and the limitations have been mentioned where generalisation cannot be implied. |
| 9 | Ethics Have ethical issues been addressed and confidentiality respected? | Yes, although ethical clearance was not required, ethical considerations have been made while using NPR’s individual level data. |

| A | Source |  |
| --- | --- | --- |
|  | Strengths and weaknesses | **Strengths**- access to data on the exact starting date of the MAUs, which made it possible to utilize the sequential implementation to isolate the effects on readmissions and mortality. Steps to ensure internal validity have been taken and the analyses account for municipal heterogeneity and external shocks trough unit/entity and time fixed effects. The results in this study are not limited to specific municipal characteristics such as municipal size or structural factors that are specific for a given municipality since this is a large-n study that utilized weighted fixed effects regression.  **Weakness**- there is a limitation in terms of generalisations of the effects if MAUs in the smallest municipalities due to some missing data from those municipalities.  Some other limitations mentioned were: short time period under study, the use of municipal aggregated as opposed to individual registry data which means that some of the effects of MAU on the outcome might not explained with the variables in the study but broader changes to the healthcare system |
|  | Source type | Quantitative study (using panel data from national registry) |
|  | Aim | to investigate whether the implementation of MAWs had a effect on reduced mortality and fewer hospital readmissions |
| B | Topic |  |
|  | Category | Norwegian hospitals and municipalities |
|  | Location(s) | Norwegian hospitals and MAWs (until 2016) |
|  | Description of IC type | Hospital admission avoidance acute in-patient intermediate care |
| C | Research methods |  |
|  | Data sample and collection | Population level data from national registry data from 2010 to 2016 have been used which means that there was no sampling was required. |
|  | Participants | All the patients who received specialist health service between 2010 and 2016. |
|  | Analysis | Fixed effect regression analysis was used to find out the effect of introduction of MAUs on mortality rates and hospital readmissions. Descriptive statistics were presented along with the regression analysis. |
|  | Time of follow-up | NA |
| D | Evidence about programme theory# | **PT11**- The association between MAU and reduction in mortality rates and hospital readmission rates are only statistically significant if the MAUs is organized as larger medical units, with 24/7 physician access to physicians.  It is only the large MAUs located at local hospitals with dedicated 24/7 physician coverage that is associated with a reduction in mortality, as they can provide high levels of specialized care |

1. **Author: Julia Crilly et al., 2011**

| 1 | Question/objective Is the research/objective question clear? | The objective of the research was to describe and evaluate the structures and processes involved in a hospital in the Nursing Home programme. |
| --- | --- | --- |
| 2 | Study design Is the study design appropriate to answer the question? | A qualitative evaluation of nurse-led HINH programme using semi structured interviews with 19 stakeholders. |
| 3 | Context Is the context or setting adequately described? | Yes, the setting of a pilot programme HINH in a aged-care facility funded by the Queensland Aged Cre reform unit taken over by Queensland health where the residents received hospital like care in the ACF. |
| 4 | Sampling Is the sample adequate to explore the range of subjects and settings? Has it been drawn from an appropriate population? | Studied only on ACF, more like a case study. |
| 5 | Data collection Was the data collection adequately described? Was it rigorously conducted to ensure confidence in the findings? | Detailed data collection has been described under the section data collection and method in page 3. |
| 6 | Data analysis Was there evidence that the data analysis was rigorously conducted to ensure confidence in the findings? | Deductive qualitative analysis of the data was done. However, some codes were revised during the data analysis. |
| 7 | Reflexivity Are the findings substantiated by the data and has consideration been given to any limitations of the methods or data that may have affected the results? | The findings are substantiated by the data but threats of credibility, confirmability, dependability and transferability occur when semi-structured questionnaires are used. However, they have tried to tackle this problem using different measures mentioned under rigour in page 3. |
| 8 | Generalisability Do any claims to generalisability follow logically and theoretically from the data? | There is no mentioning of generalisability and the authors mention that the findings might not be transferrable to other units with different structure and organisation. |
| 9 | Ethics Have ethical issues been addressed and confidentiality respected? | This research was approved by the University and Health Service District Human Research Ethics Committees. All  participants gave written informed consent to interview |
| A | Source |  |
|  | Strengths and weaknesses | **Strengths-** Measures to tackle methodological limitations has been taken.The Hospital in the Nursing Home programme (led by a nurse) can address the gap in the care pathway between aged-care facility and hospital for some acutely unwell older adults. Structural and process evaluation of a programme. The participants include variety of stakeholders which is hoped to be a representative sample.  **Weakness**- the sample size was limited in terms of number of participants and to one regional healthcare district in Australia. A limitation exists with using a priori codes for data analysis. This approach may have impacted on the study’s findings in terms of reliability (stability and reproducibility of coding) and validity. |
|  | Source type | Qualitative data collected using semi-structured questionnaires provided to key stakeholders like GPs, nurses, ACF residents, programme manager, etc. |
|  | Aim | The objective of the research was to describe and evaluate the structures and processes involved in a hospital in the Nursing Home programme. |
| B | Topic |  |
|  | Category | 17 service providers from and 2 ACF resident HINH in Aged care facilities (5 of the 36 ACFs in the region) |
|  | Location(s) | Queensland region in Australia |
|  | Description of IC type | HINH provided in ACFs to the residents |
| C | Research methods |  |
|  | Data sample and collection | A sample of 19 participants representing 5 of 36 ACFs in Queensland health region. Qualitative data collection using semi-structured questionnaire. |
|  | Participants | 10 ACF staff, three HINH healthcare professionals, two GPs, two ED nurses, and two residents |
|  | Analysis | Deductive analysis of qualitative data |
|  | Time of follow-up | NA |
| D | Evidence about programme theory# | #PT2- These structures were identified as imperative to programme utilization. For example, with the social structure of responsibility for service provision, there was an understanding that the clinical care of the ACF resident if in ED or hospital was by hospital staff, but when discharged from the ED or hospital back to the ACF, clinical responsibility resided with the GP. P7,c1  #PT3- GP’s found that when the HINH programme manager provided additional assessment of an ACF resident at risk of hospital admission, this was beneficial. One GP stated that had it not been available, the ACF resident would most certainly need to be transferred to hospital: Advantages for GPs is that … we can care more for our residents in the nursing homes with those added services [HINH] (GP 2). P7,c2 The role of the HINH programme also involved service  development and expansion and care coordination. As a result of the workshop and on-site education, the acute care  skills of ACF nurses developed. This resulted in hospital avoidance for some ACF residents. P7, c2  The HINH programme manager’s role involved coordinating care by liaising with and referring ACF residents to  specialist services. This process was done in collaboration with the ACF resident’s GP. This worked well as the HINH  programme manager had a background in both acute care nursing in the hospital and community-based nursing and  thus was not only aware of service options available, but had a pre-established working relationship with some of the other service providers. By organizing and bringing together additional resources (such as allied health, pathology,  specialist medical and nursing practitioners, as appropriate) the patients could receive some treatment in the ACF p8, c2  Miscommunication between the GP and the HINH programme may have led to some ACF residents being transferred to the ED and others transferred back to the ACF with limited feedback or support |
