# Supplementary material for: Providing effective and efficient hospital admission avoidance inpatient care: a systematic realist review of Norwegian municipal inpatient acute care services
Source: BMC Health Serv Res. 2026 Apr 29;26:834. doi: 10.1186/s12913-026-14621-z (PMC13270756; doi:10.1186/s12913-026-14621-z)
Supplement: Supplementary file 2 — Supplementary Material 2: Explanation of methodology-Detailed systematic strategy used to search for articles [file 12913_2026_14621_MOESM2_ESM.docx]

**Database searches**

## Stage 1

Search for literature to formulate/locate initial programme theories was done in two iterations. The first iteration included searching in Medline which generated 2289 hits. This was followed by a second search in Medline with different keywords which generated 81 hits. Similarly, searches were also carried out in Embase and CINAHL that generated a total of 371 unique literature. All the literature thus obtained were screened. The database search was carried out primarily by Simon Briscoe with inputs from Rob Anderson, Marianne Sundlisæter Skinner and Sujan Rijal.

### 1^st^ iteration search

Database: MEDLINE
Host: Ovid
Data Parameters: 1946 to November 08, 2022
Date Searched: 9/11/2022
Searcher: SB
Hits: 2289
Strategy:

1. ((district* or local* or municipal*) adj3 acute adj3 (care or healthcare or unit* or ward*)).tw,kw.
2. MIPAC.tw,kw.
3. (decentrali?ed adj3 acute adj3 (care or healthcare or unit* or ward*)).tw,kw.
4. ((district* or local* or municipal*) adj3 emergency adj3 (bed* or care or healthcare or unit* or ward*)).tw,kw.
5. ((hospit* or "intermediate care" or "primary care") adj4 admission* adj4 (avoidance or avoidable)).tw.
6. or/1-5
7. ((community or cottage) adj2 hospital*).tw.
8. Hospitals, Community/
9. Intermediate Care Facilities/
10. "ambulatory care sensitive".tw.
11. or/7-10
12. ((evaluation or pilot or validation) adj2 (studies or study)).tw.
13. ("evaluation studies" or "validation studies").pt.
14. evaluation studies as topic/
15. Pilot projects/
16. program evaluation/
17. validation studies as topic/
18. (program* adj6 evaluat*).ti,ab.
19. or/12-18
20. 11 and 19
21. 6 or 20

### 2^nd^ iteration searches

Database: MEDLINE
Host: Ovid
Data Parameters: 1946 to May 08, 2023
Date Searched: 9/5/2023
Searcher: SB
Hits: 81
Strategy:

1. "Acute care unit*".tw,kw.
2. (denmark or danish or copenhagen or aarhus or odense or aalborg or esbjerg or randers or kolding or horsens or vejle or roskilde or Hovedstaden or Midtjylland or Nordjylland or zealand or Syddanmark).tw,kw.
3. Denmark/
4. 2 or 3
5. 1 and 4
6. "acute medical assessment unit*".tw,kw
7. ("medical assessment" adj2 "planning unit*").tw,kw
8. "acute assessment unit*".tw,kw
9. "acute planning unit*".tw,kw
10. "rapid assessment medical unit*".tw,kw
11. "early assessment medical unit*".tw,kw
12. or/5-11

Database: Embase
Host: Ovid
Data Parameters: 1974 to 2023 May 08
Date Searched: 9/5/2023
Searcher: SB
Hits: 315
Strategy:

1. ((district* or local* or municipal*) adj3 acute adj3 (care or healthcare or unit* or ward*)).tw,kw.
2. MIPAC.tw,kw.
3. "Acute care unit*".tw,kw.
4. (denmark or danish or copenhagen or aarhus or odense or aalborg or esbjerg or randers or kolding or horsens or vejle or roskilde or Hovedstaden or Midtjylland or Nordjylland or zealand or Syddanmark).tw,kw.
5. Denmark/
6. 4 or 5
7. 3 and 6
8. "acute medical assessment unit*".tw,kw.
9. ("medical assessment" adj2 "planning unit*").tw,kw.
10. "acute assessment unit*".tw,kw.
11. "acute planning unit*".tw,kw.
12. "rapid assessment medical unit*".tw,kw.
13. "early assessment medical unit*".tw,kw.
14. or/1-2,7-13

Database: CINAHL
Host: EBSCO
Data Parameters: n/a
Date Searched: 9/5/2023
Searcher: SB
Hits: 142
Strategy:

1. TI ( ((district* or local* or municipal*) N2 acute N2 (care or healthcare or unit* or ward*)) ) OR AB ( ((district* or local* or municipal*) N2 acute N2 (care or healthcare or unit* or ward*)) )
2. TI MIPAC OR AB MIPAC
3. TI "Acute care unit*" OR AB "Acute care unit*"
4. TI "acute medical assessment unit*" OR AB "acute medical assessment unit*"
5. TI ("medical assessment" N1 "planning unit*") OR AB ("medical assessment" N1 "planning unit*")
6. TI "acute assessment unit*" OR AB "acute assessment unit*"
7. TI "acute planning unit*" OR AB "acute planning unit*"
8. TI "rapid assessment medical unit*" OR AB "rapid assessment medical unit*"
9. TI "early assessment medical unit*" OR AB "early assessment medical unit*"
10. TI ( (denmark or danish or copenhagen or aarhus or odense or aalborg or esbjerg or randers or kolding or horsens or vejle or roskilde or Hovedstaden or Midtjylland or Nordjylland or Sjælland or Syddanmark) ) OR AB ( (denmark or danish or copenhagen or aarhus or odense or aalborg or esbjerg or randers or kolding or horsens or vejle or roskilde or Hovedstaden or Midtjylland or Nordjylland or Sjælland or Syddanmark) )
11. (MH "Denmark")
12. S10 OR S11
13. S3 AND S12
14. S1 OR S2 OR S4 OR S5 OR S6 OR S7 OR S8 OR S9 OR S13

Table X. Bibliographic database search results

| **Database** | **Hits** |
| --- | --- |
| MEDLINE |  |
| Embase |  |
| CINAHL |  |
| **Total records** | **538** |
| **Duplicate records** |  |
| **Unique records** | **371** |

The search was later extended to January 2026 where 336 new studies were retrieved.

| **#** | **Searches** | **Results** |
| --- | --- | --- |
| 1 | ((district* or local* or municipal*) adj3 acute adj3 (care or healthcare or unit* or ward*)).tw,kw. | 114 |
| 2 | MIPAC.tw,kw. | 11 |
| 3 | (decentrali?ed adj3 acute adj3 (care or healthcare or unit* or ward*)).tw,kw. | 10 |
| 4 | ((district* or local* or municipal*) adj3 emergency adj3 (bed* or care or healthcare or unit* or ward*)).tw,kw. | 109 |
| 5 | ((hospit* or "intermediate care" or "primary care") adj4 admission* adj4 (avoidance or avoidable)).tw. | 374 |
| 6 | or/1-5 | 607 |
| 7 | ((community or cottage) adj2 hospital*).tw. | 28301 |
| 8 | Hospitals, Community/ | 12446 |
| 9 | Intermediate Care Facilities/ | 740 |
| 10 | "ambulatory care sensitive".tw. | 1027 |
| 11 | or/7-10 | 36250 |
| 12 | ((evaluation or pilot or validation) adj2 (studies or study)).tw. | 197909 |
| 13 | ("evaluation studies" or "validation studies").pt. | 26 |
| 14 | evaluation studies as topic/ | 122495 |
| 15 | Pilot projects/ | 165398 |
| 16 | program evaluation/ | 70123 |
| 17 | validation studies as topic/ | 2478 |
| 18 | (program* adj6 evaluat*).ti,ab. | 60423 |
| 19 | or/12-18 | 498661 |
| 20 | 11 and 19 | 1943 |
| 21 | 6 or 20 | 2547 |
| 22 | limit 21 to yr="2023 -Current" | 336 |

## Stage 2

Stage 2 search was carried out in three databases similar to Stage 1. Pertaining to the lack of rich evidence for testing programme theories, a country specific search approach was undertaken. Schemes that were similar to Norwegian MIPACs in two components (in-patient and designed for hospital admission avoidance) was listed out for all the high-income European countries (wherever possible).

Database: MEDLINE
Host: Ovid
Data Parameters:
Date Searched:
Searcher: SB
Hits:
Strategy:

| **#** | **Searches** | **Country** |
| --- | --- | --- |
| 1 | "municipal health cent*".tw,kw. | Finland and Sweden |
| 2 | "primary health care cent*".tw. | Spain |
| 3 | (spain or spanish or madrid* or barcelona* or valencia* or seville* or bilbao* or malaga* or "oviedo–gijón–avilés*" or alicante* or "las palmas*" or zaragoza* or murcia* or cadiz* or biscay* or "a coruna*").tw,kw. |  |
| 4 | Spain/ |  |
| 5 | 3 or 4 |  |
| 6 | 2 and 5 |  |
| 7 | (nurs* adj1 "led hospital*").tw,kw. | Australia and New Zealand |
| 8 | "acute demand management service*".tw,kw. |  |
| 9 | "acute medical unit*".tw,kw. |  |
| 10 | (australia* or melbourne* or sydney* or brisbane* or perth* or adelaide* or "gold coast*" or "newcastle-maitland*" or canberra* or "sunshine coast" or "central coast*" or "new south wales*" or victoria* or queensland* or "western australia*" or "south australia*" or tasmania*).tw,kw. |  |
| 11 | exp Australia/ |  |
| 12 | 10 or 11 |  |
| 13 | ("new zealand*" or auckland* or christchurch* or wellington* or hamilton* or tauranga* or "lower hutt*" or dunedin* or "palmerston north*" or napier* or porirua* or canterbury* or waikato* or "bay of plenty*" or "manawatu-whanganui" or otago* or northland* or "hawke's bay*" or taranaki*).tw,kw. |  |
| 14 | New Zealand/ |  |
| 15 | 13 or 14 |  |
| 16 | 12 or 15 |  |
| 17 | 9 and 16 |  |
| 18 | 7 or 8 or 17 |  |
| 19 | "acute medical ward*".tw,kw. | UK |
| 20 | exp United Kingdom/ |  |
| 21 | (national health service* or nhs*).ti,ab,in. |  |
| 22 | (english not ((published or publication* or translat* or written or language* or speak* or literature or citation*) adj5 english)).ti,ab. |  |
| 23 | (gb or "g.b." or britain* or (british* not "british columbia") or uk or "u.k." or united kingdom* or (england* not "new england") or northern ireland* or northern irish* or scotland* or scottish* or ((wales or "south wales") not "new south wales") or welsh*).ti,ab,jw,in. |  |
| 24 | (bath or "bath's" or ((birmingham not alabama*) or ("birmingham's" not alabama*) or bradford or "bradford's" or brighton or "brighton's" or bristol or "bristol's" or carlisle* or "carlisle's" or (cambridge not (massachusetts* or boston* or harvard*)) or ("cambridge's" not (massachusetts* or boston* or harvard*)) or (canterbury not zealand*) or ("canterbury's" not zealand*) or chelmsford or "chelmsford's" or chester or "chester's" or chichester or "chichester's" or coventry or "coventry's" or derby or "derby's" or (durham not (carolina* or nc)) or ("durham's" not (carolina* or nc)) or ely or "ely's" or exeter or "exeter's" or gloucester or "gloucester's" or hereford or "hereford's" or hull or "hull's" or lancaster or "lancaster's" or leeds* or leicester or "leicester's" or (lincoln not nebraska*) or ("lincoln's" not nebraska*) or (liverpool not (new south wales* or nsw)) or ("liverpool's" not (new south wales* or nsw)) or ((london not (ontario* or ont or toronto*)) or ("london's" not (ontario* or ont or toronto*)) or manchester or "manchester's" or (newcastle not (new south wales* or nsw)) or ("newcastle's" not (new south wales* or nsw)) or norwich or "norwich's" or nottingham or "nottingham's" or oxford or "oxford's" or peterborough or "peterborough's" or plymouth or "plymouth's" or portsmouth or "portsmouth's" or preston or "preston's" or ripon or "ripon's" or salford or "salford's" or salisbury or "salisbury's" or sheffield or "sheffield's" or southampton or "southampton's" or st albans or stoke or "stoke's" or sunderland or "sunderland's" or truro or "truro's" or wakefield or "wakefield's" or wells or westminster or "westminster's" or winchester or "winchester's" or wolverhampton or "wolverhampton's" or (worcester not (massachusetts* or boston* or harvard*)) or ("worcester's" not (massachusetts* or boston* or harvard*)) or (york not ("new york*" or ny or ontario* or ont or toronto*)) or ("york's" not ("new york*" or ny or ontario* or ont or toronto*))))).ti,ab,in. |  |
| 25 | (bangor or "bangor's" or cardiff or "cardiff's" or newport or "newport's" or st asaph or "st asaph's" or st davids or swansea or "swansea's").ti,ab,in. |  |
| 26 | (aberdeen or "aberdeen's" or dundee or "dundee's" or edinburgh or "edinburgh's" or glasgow or "glasgow's" or inverness or (perth not australia*) or ("perth's" not australia*) or stirling or "stirling's").ti,ab,in. |  |
| 27 | (armagh or "armagh's" or belfast or "belfast's" or lisburn or "lisburn's" or londonderry or "londonderry's" or derry or "derry's" or newry or "newry's").ti,ab,in. |  |
| 28 | or/20-27 |  |
| 29 | (exp africa/ or exp americas/ or exp antarctic regions/ or exp arctic regions/ or exp asia/ or exp australia/ or exp oceania/) not (exp United Kingdom/ or europe/) |  |
| 30 | 28 not 29 |  |
| 31 | 19 and 30 |  |

Database: Embase
Host: Ovid
Data Parameters:
Date Searched:
Searcher: SB
Hits:
Strategy:

| **#** | **Searches** | **Country** |
| --- | --- | --- |
| 1 | "municipal health cent*".tw,kw. | Finland and Sweden |
| 2 | "primary health care cent*".tw,kw. | Spain |
| 3 | (spain or spanish or madrid* or barcelona* or valencia* or seville* or bilbao* or malaga* or "oviedo–gijón–avilés*" or alicante* or "las palmas*" or zaragoza* or murcia* or cadiz* or biscay* or "a coruna*").tw,kw. |  |
| 4 | exp Spain/ |  |
| 5 | 3 or 4 |  |
| 6 | 2 and 5 |  |
| 7 | (nurs* adj1 "led hospital*").tw,kw. | Australia and New Zealand |
| 8 | "acute demand management service*".tw,kw. |  |
| 9 | "acute medical unit*".tw,kw. |  |
| 10 | (australia* or melbourne* or sydney* or brisbane* or perth* or adelaide* or "gold coast*" or "newcastle-maitland*" or canberra* or "sunshine coast" or "central coast*" or "new south wales*" or victoria* or queensland* or "western australia*" or "south australia*" or tasmania*).tw,kw. |  |
| 11 | ("new zealand*" or auckland* or christchurch* or wellington* or hamilton* or tauranga* or "lower hutt*" or dunedin* or "palmerston north*" or napier* or porirua* or canterbury* or waikato* or "bay of plenty*" or "manawatu-whanganui" or otago* or northland* or "hawke's bay*" or taranaki*).tw,kw. |  |
| 12 | exp "Australia and New Zealand"/ |  |
| 13 | or/10-12 |  |
| 14 | 9 and 13 |  |
| 15 | 7 or 8 or 14 |  |
| 16 | "acute medical ward*".tw,kw. | UK |
| 17 | exp United Kingdom/ |  |
| 18 | (national health service* or nhs*).ti,ab,in,ad. |  |
| 19 | (english not ((published or publication* or translat* or written or language* or speak* or literature or citation*) adj5 english)).ti,ab. |  |
| 20 | (gb or "g.b." or britain* or (british* not "british columbia") or uk or "u.k." or united kingdom* or (england* not "new england") or northern ireland* or northern irish* or scotland* or scottish* or ((wales or "south wales") not "new south wales") or welsh*).ti,ab,jx,in,ad. |  |
| 21 | (bath or "bath's" or ((birmingham not alabama*) or ("birmingham's" not alabama*) or bradford or "bradford's" or brighton or "brighton's" or bristol or "bristol's" or carlisle* or "carlisle's" or (cambridge not (massachusetts* or boston* or harvard*)) or ("cambridge's" not (massachusetts* or boston* or harvard*)) or (canterbury not zealand*) or ("canterbury's" not zealand*) or chelmsford or "chelmsford's" or chester or "chester's" or chichester or "chichester's" or coventry or "coventry's" or derby or "derby's" or (durham not (carolina* or nc)) or ("durham's" not (carolina* or nc)) or ely or "ely's" or exeter or "exeter's" or gloucester or "gloucester's" or hereford or "hereford's" or hull or "hull's" or lancaster or "lancaster's" or leeds* or leicester or "leicester's" or (lincoln not nebraska*) or ("lincoln's" not nebraska*) or (liverpool not (new south wales* or nsw)) or ("liverpool's" not (new south wales* or nsw)) or ((london not (ontario* or ont or toronto*)) or ("london's" not (ontario* or ont or toronto*)) or manchester or "manchester's" or (newcastle not (new south wales* or nsw)) or ("newcastle's" not (new south wales* or nsw)) or norwich or "norwich's" or nottingham or "nottingham's" or oxford or "oxford's" or peterborough or "peterborough's" or plymouth or "plymouth's" or portsmouth or "portsmouth's" or preston or "preston's" or ripon or "ripon's" or salford or "salford's" or salisbury or "salisbury's" or sheffield or "sheffield's" or southampton or "southampton's" or st albans or stoke or "stoke's" or sunderland or "sunderland's" or truro or "truro's" or wakefield or "wakefield's" or wells or westminster or "westminster's" or winchester or "winchester's" or wolverhampton or "wolverhampton's" or (worcester not (massachusetts* or boston* or harvard*)) or ("worcester's" not (massachusetts* or boston* or harvard*)) or (york not ("new york*" or ny or ontario* or ont or toronto*)) or ("york's" not ("new york*" or ny or ontario* or ont or toronto*))))).ti,ab,in,ad. |  |
| 22 | (bangor or "bangor's" or cardiff or "cardiff's" or newport or "newport's" or st asaph or "st asaph's" or st davids or swansea or "swansea's").ti,ab,in,ad. |  |
| 23 | (aberdeen or "aberdeen's" or dundee or "dundee's" or edinburgh or "edinburgh's" or glasgow or "glasgow's" or inverness or (perth not australia*) or ("perth's" not australia*) or stirling or "stirling's").ti,ab,in,ad. |  |
| 24 | (armagh or "armagh's" or belfast or "belfast's" or lisburn or "lisburn's" or londonderry or "londonderry's" or derry or "derry's" or newry or "newry's").ti,ab,in,ad. |  |
| 25 | or/17-24 |  |
| 26 | (exp "arctic and antarctic"/ or exp oceanic regions/ or exp western hemisphere/ or exp africa/ or exp asia/) not (united kingdom/ or europe/) |  |
| 27 | 25 not 26 |  |
| 28 | 16 and 27 |  |

Database: CINAHL
Host: EBSCO
Data Parameters:
Date Searched:
Searcher: SB
Hits:
Strategy:

| **#** | **Searches** | **Country** |
| --- | --- | --- |
| S1 | TI "municipal health cent*" OR AB "municipal health cent*" | Finland and Sweden |
| S2 | TI "primary health care cent*" OR AB "primary health care cent*" | Spain |
| S3 | TI ( (spain or spanish or madrid* or barcelona* or valencia* or seville* or bilbao* or malaga* or "oviedo–gijón–avilés*" or alicante* or "las palmas*" or zaragoza* or murcia* or cadiz* or biscay* or "a coruna*") ) OR AB ( (spain or spanish or madrid* or barcelona* or valencia* or seville* or bilbao* or malaga* or "oviedo–gijón–avilés*" or alicante* or "las palmas*" or zaragoza* or murcia* or cadiz* or biscay* or "a coruna*") ) |  |
| S4 | (MH "Spain") |  |
| S5 | S3 OR S4 |  |
| S6 | S2 AND S5 |  |
| S7 | TI nurs* N0 "led hospital*" OR AB nurs* N0 "led hospital*" | Australia and New Zealand |
| S8 | TI "acute medical unit*" OR AB "acute medical unit*" |  |
| S9 | TI ( (australia* or melbourne* or sydney* or brisbane* or perth* or adelaide* or "gold coast*" or "newcastle-maitland*" or canberra* or "sunshine coast" or "central coast*" or "new south wales*" or victoria* or queensland* or "western australia*" or "south australia*" or tasmania*) ) OR AB ( (australia* or melbourne* or sydney* or brisbane* or perth* or adelaide* or "gold coast*" or "newcastle-maitland*" or canberra* or "sunshine coast" or "central coast*" or "new south wales*" or victoria* or q [...](javascript:showHistoryTerm('ctl00_ctl00_FindField_FindField_historyControl_HistoryRepeater_ctl08_ellipsis',true)) |  |
| S10 | TI ( ("new zealand*" or auckland* or christchurch* or wellington* or hamilton* or tauranga* or "lower hutt*" or dunedin* or "palmerston north*" or napier* or porirua* or canterbury* or waikato* or "bay of plenty*" or "manawatu-whanganui" or otago* or northland* or "hawke's bay*" or taranaki*) ) OR AB ( ("new zealand*" or auckland* or christchurch* or wellington* or hamilton* or tauranga* or "lower hutt*" or dunedin* or "palmerston north*" or napier* or porirua* or canterbury* or waikato* or "bay [...](javascript:showHistoryTerm('ctl00_ctl00_FindField_FindField_historyControl_HistoryRepeater_ctl09_ellipsis',true)) |  |
| S11 | (MH "Australia+") |  |
| S12 | (MH "New Zealand") |  |
| S13 | S9 OR S10 OR S11 OR S12 |  |
| S14 | S8 AND S13 |  |
| S15 | TI "acute demand management service*" OR AB "acute demand management service*" |  |
| S16 | S7 OR S14 OR S15 |  |
| S17 | TI "acute medical ward*" OR AB "acute medical ward*" | UK |

Table X. Bibliographic database search results

| Database | Hits |
| --- | --- |
| Finland/Sweden |  |
| MEDLINE | 135 |
| Embase | 140 |
| CINAHL | 56 |
| **Total** | **331** |
| **Duplicate** | **149** |
| **Unique** | **182** |
| Spain |  |
| MEDLINE | 324 |
| Embase | 370 |
| CINAHL | 137 |
| **Total** | **831** |
| **Duplicate** | **371** |
| **Unique** | **460** |
| Australia/New Zealand |  |
| MEDLINE | 36 |
| Embase | 65 |
| CINAHL | 36 |
| **Total** | **137** |
| **Duplicate** | **58** |
| **Unique** | **79** |
| UK |  |
| MEDLINE | 114 |
| Embase | 206 |
| CINAHL | 194 |
| **Total** | **514** |
| **Duplicate** | **171** |
| **Unique** | **343** |

**Screening**

Our aim through this screening process was to identify literature that had some explanation of in-patient hospital admission avoidance. As mentioned in the protocol, we used some inclusion criteria to screen the studies. We basically had three criteria for the intermediate care scheme to be considered eligible for inclusion i.e., a) In-patient care, b) admission avoidance, and c)had to be managed at the community level. All the intermediate care schemes that fulfilled only two of the three criteria were excluded as it was out of scope for this review. However, this inclusion criteria were set as a guideline rather than fixed set of rules with possibility of amendment if seen necessary during the screening process. The studies from 2022, 2021 and 2020 were screened by three reviewers independently (Sujan Rijal, Rob Anderson and Fan Yang) to ensure uniform understanding of the criteria and to eliminate any form of bias. We compared the result of independent screening and discussed the decisions made. Rest of the screening was done by SR and FY independently.

**Identifying programme theories from sources**

Definitions of ‘programme theory’ originate from the American ‘theory-driven evaluation’ community. Whilst not uniform, these definitions share an understanding of a programme theory as a proposition for how a programme is supposed to produce intended outcomes ([Chen, 1990](#_ENREF_1), [Rossi et al., 2004](#_ENREF_9), [Weiss, 1998](#_ENREF_15)). Twenty-nine studies were selected from screening for full text reading out of which only eleven were deemed theoretically rich enough to formulate initial programme theories. We adopted if-then-because approach of identifying programme theories in the literature. We primarily searched in results and discussion section of the articles. Statements were broken down into if (contexts)- then (outcomes) – because (mechanisms). Such findings were listed in an Excel file for each statement assumed to mention something about why and how AAIC services were thought to function. Statements were extracted from one study at a time which was easy to follow and reference at the end. Again, to maintain uniformity, SR and RA read through three studies independently and extracted information about programme theories. We went through our results and discussed if one had missed anything that the other person has managed to capture. The rest of extraction process was conducted by SR. After the initial process of listing possible programme theories, statements regarded as non-explanatory and non-causal were excluded. Statements that were similar (with similar context or/and mechanism) were merged together to make one single statement. List of programme theories from each stage of formulation has been documented in excel files provided separately in the supporting files. The final preliminary list of programme theories was then grouped according to the intended outcomes that were then sent for stakeholder consultation to assess the logic, plausibility, relevance, and explanatory power. They were also asked to provide comments if they thought any further edit was necessary to make the programme theories clearer. After incorporating feedback from the stakeholders, programme theories that were specific to MIPAC and Norwegian context was written in more generic terms. Seven programme theories were listed and subjected to evidence testing.

Example of ‘If…Then..Because’ propositions to develop conceptual framework

| If | Then | Because | Location | Remarks |
| --- | --- | --- | --- | --- |
| AAIC services are staffed with professional nurses and doctors | patients felt safe and the perceived quality of treatment was comparable to hospitals | personnel in AAIC units have the ability to observe and evaluate each patient's condition continuously and take necessary action | p320, c1 | Staffing and patient experience |
| AAIC service units have limited diagnostic and treatment options, and competence | patients are sceptic about the quality of the services provided | they value being diagnosed faster than abstract things like kindness | p321, c1, top | Patient experience with facilities |
| AAIC services are located closer to home | patients are more satisfied with the care | they do not have travel to the hospital which is away from home and it is easier for the relatives to come and visit | p321, c1, mid | Geographical location |
| AAIC services have single rooms for each patient | patients are more satisfied with the care | it enables self-chosen seclusion or socialising with other patients, more opportunities to relax and more privacy | p321, c2, top | Physical environment and patient satisfaction |
| AAIC has enough competent staffs to look after the patients | they feel more secure and satisfied with the care | they do not have to go through long waiting times, staffs have enough time observe and follow up each patient, and respond quickly whenever needed, partly also because the workload in AAIC units is lower than hosptials | p321, c2, mid-bot | Waiting times and patient satisfaction |
