# Supplementary material for: Providing effective and efficient hospital admission avoidance inpatient care: a systematic realist review of Norwegian municipal inpatient acute care services
Source: BMC Health Serv Res. 2026 Apr 29;26:834. doi: 10.1186/s12913-026-14621-z (PMC13270756; doi:10.1186/s12913-026-14621-z)
Supplement: Supplementary file 4 — Supplementary Material 4: Review protocol- protocol submitted to PROSPERO before conducting the review [file 12913_2026_14621_MOESM4_ESM.docx]

**Review protocol**

**Evaluating and explaining the effectiveness (and costs) of hospital admission avoidance in-patient intermediate care schemes in high-income: a realist review**

**Realist review: why and how?**

Every intervention has an underlying theory which suggests the ways in which the intervention should be implemented to achieve improvement gains as outcomes (Pawson et al., 2005). It is optimal if they are designed in accordance with these underlying theories, more so for complex interventions. Inspection of intermediate outputs for successful achievement of outcomes, and considerations that interventions are dynamic, non-linear, and embedded in multiple layers of social system structure are vital in the evaluation of healthcare interventions (Anderson, 2008). Many health interventions, moreover, require active participation from users and/or providers as stakeholders’ knowledge is integral to better understand the implementation and effectiveness of the intervention.

Systematic review method aims to assess the effectiveness of an intervention by collecting empirical evidence from previous studies and provide a generalisable answer that applies to all types of setting. The data so gathered are analysed to produce an idea if the intervention works or produce an average estimate of effect of an intervention. For instance, a systematic review assesses the effect of intervention A on a specific health issue (in comparison to intervention B) using all the studies that have assessed the effectiveness of intervention A (and B) and conclude whether A is (or not) effective (additionally compared to B).

However, complex interventions as mentioned above are dynamic in that they self-transform and are never implemented quite the same way every time (Pawson et al., 2005, Pearson et al., 2013). Such universal or generalisable conclusion from systematic reviews are not reliable and might not represent the real scenario. Moreover, such reviews do not usually explain the why of the effectiveness of the intervention. Such one-dimensional explanation of interventions rarely is helpful in policy making.

Realist review of such complex interventions attempt to address these limitations by recognizing (broadly) that:

- An intervention that works in one setting might not work in another setting. Every setting represents unique underlying contexts and mix of components. Thus, interventions do not always produce a generalisable effect.
- Success of interventions are also based on the mechanisms which enable or support the improved outcomes. Thus, it is not the intervention itself that produces the effect but various enabling/disabling mechanisms which produce intermediate changes towards a bigger outcome.

Thus, asking questions of what works, for whom and under circumstances are essential to understanding the complexities of these interventions, and giving a potential context-specific recommendations instead of seeking to make universally applicable judgements or conclusion (Berg and Nanavati, 2016).

**Background and rationale for the review**

Intermediate care is a label for a range of schemes that have emerged in the last few decades in the attempt to provide care for those who would otherwise need to be admitted to hospital or stay in hospital longer (Pearson et al., 2015, Young, 2009). They are care or service(s) concerned with transition of patients from hospital to home helping them achieve functional independence from medical/social dependence (Steiner, 2001). Various models of intermediate care have been adopted by health systems around the world. Community hospitals, hospital-at-home, supported early discharge schemes, community care centres, hostel beds/patient hotel, in-patient nursing beds and General Practitioner (GP) nursing home beds are some examples. Such schemes have been helpful to either enable early discharge of patients, reducing the burden on specialist hospital services in terms of workload and costs, or maintain care continuity at the primary level by providing care to people closer to their home (Sezgin et al., 2020, Coleman and Boult, 2003, WHO, 2016).

For decades, research has focused on the post-discharge models and their effectiveness (Pearson et al., 2013, Swanson and Hagen, 2016). Admission avoidance intermediate care models have not been as widely discussed or evaluated. Avoidable hospital admissions are one of the most important drivers for pressures on acute hospital care, because they involve patients who do not require urgent attention or complex interventions and could otherwise have been treated in a less specialized unit (Hoot and Aronsky, 2008).

One model of admission avoidance intermediate care, Municipal In-Patient Acute Care (MIPAC), was introduced in Norway as a part of the Coordination Reform in 2012 (Vatnøy et al., 2020). It had the explicit aim of reducing pressure on specialist care services and providing patients with services closer to home. However, there have been various issues with MIPAC like lack of trust of the service by GPs (Nystrøm et al., 2022); incompetent coordination between GPs, hospital physicians, and nurses (Johannessen and Steihaug, 2020); lack of clarity in patient responsibility (Nystrøm et al., 2021), etc., which have resulted in unsatisfactory level of bed occupancy even though huge amount of state and municipal resources are being used (Skinner, 2015).

Although the government has been successful in transferring healthcare service delivery to local/municipal level, with aforementioned issues and significantly lower utilisation rates than aimed for, more research on why MIPAC units are not able to achieve the set goals is necessary. More so with huge resources being spent by the state and municipalities to deliver this health care service.

Although various hospital admission avoidance intermediate care models are prevalent in the world and Europe, there are no studies to our knowledge that have assessed their effectiveness. Keeping in mind these models have been implemented in different contexts, a realist review to understand the context and mechanisms which affect the effectiveness of these models in a particular scenario would be beneficial in informing policy makers about underlying factors of effectiveness (lack thereof) of models like MIPAC.

This review is a part of a larger research project funded by the Research Council of Norway: Variations in trajectories, utilisation, and costs in Norwegian municipal in-patient acute care units (project number: 324959) (RCN, 2021). Apart from synthesising evidence on admission avoidance schemes, this review will also inform other work packages of the project which look at patient trajectories in MIPAC and costs of operating MIPACs in Norway.

**Review questions**

Which hospital admission avoidance in-patient intermediate care schemes work, for whom, at what cost, in what circumstances and why? Sub-questions for the realist review are:

1. What are the **different inpatient healthcare services/units as alternatives to acute inpatient hospital specialised care in high-income countries?**
2. What are the causal mechanisms that underlie **the effectiveness of hospital admission avoidance in-patient intermediate care models**?
3. What are the causal mechanisms that affect **the costs of** **hospital admission avoidance in-patient intermediate care models?**
4. What are the relevant contexts that enable (or prevent) the outcomes (cost and effectiveness) from being realised, and in relation to the identified mechanisms (from Q2 and 3)?

**Overall design**

This realist review will be conducted in several stages, using different published and unpublished sources to identify, define, and refine realist programme theories with evidence.

**Stage 1: Searching for programme theories**

This stage will search for initial programme theories. For the scope of the realist review, only those models that provide 24-hour in-patient acute care services provided outside hospitals at the local level which are lower level of care than specialised hospitals, are not step-down or early supported discharges models of care in high income countries will be included. This will initially be focused on evidence from Europe, Canada, and Australasia in order to prioritise evidence from health systems that are dominantly public funded (i.e., general taxation or social/compulsory health insurance). This will help us narrow down the scope of the review and focus on intermediate care models that have main components relevant to the review and wider research project.

The searches for existing theories will make use of (a) published and grey literature evaluations of AAIC schemes in Europe, Canada and Australasia; b) a review of evaluations of the Norwegian MipAC schemes; (c) stakeholder consultation among those managing, delivering, and evaluating the Norwegian MIPAC model. Information specialist Simon Briscoe (SB) has developed and implemented a search strategy for stage 1. The search strategy with keywords used has been listed in the table below.

| MIPAC searches |
| --- |
| 1^st^ iteration MEDLINE search |
| Database: MEDLINE Host: Ovid Data Parameters: 1946 to November 08, 2022 Date Searched: 9/11/2022  Searcher: SB Hits: 2289 Strategy: |
| 1. ((district* or local* or municipal*) adj3 acute adj3 (care or healthcare or unit* or ward*)).tw,kw. |
| 1. MIPAC.tw,kw. |
| 1. (decentrali?ed adj3 acute adj3 (care or healthcare or unit* or ward*)).tw,kw. |
| 1. ((district* or local* or municipal*) adj3 emergency adj3 (bed* or care or healthcare or unit* or ward*)).tw,kw. |
| 1. ((hospit* or "intermediate care" or "primary care") adj4 admission* adj4 (avoidance or avoidable)).tw. |
| 1. or/1-5 |
| 1. ((community or cottage) adj2 hospital*).tw. |
| 1. Hospitals, Community/ |
| 1. Intermediate Care Facilities/ |
| 1. "ambulatory care sensitive".tw. |
| 1. or/7-10 |
| 1. ((evaluation or pilot or validation) adj2 (studies or study)).tw. |
| 1. ("evaluation studies" or "validation studies").pt. |
| 1. evaluation studies as topic/ |
| 1. Pilot projects/ |
| 1. program evaluation/ |
| 1. validation studies as topic/ |
| 1. (program* adj6 evaluat*).ti,ab. |
| 1. or/12-18 |
| 1. 11 and 19 |
| 1. 6 or 20 |

This database search will be supplemented by forward and backward citation searching by the reviewers. The approach is likely to be exploratory and iterative, in contrast to the more formal searches carried out later in the review.

**Stage 2:** **Developing program theories**

This stage will involve the identification and further development of programme theories, that is, identification and explanation of probable causal mechanisms and relevant contextual factors that describe how admission avoidance intermediate care models achieve their outcomes (can be positive or negative outcomes). This will be done through the review of available literature from Stage 1 and stakeholder’s meeting.

**Stage 3**: **Refining programme theories**

This is the final stage of the review and includes refining the developed programme theories with empirical evidence. We will refine selected programme theories (that is, particular combinations of causal mechanisms, related contexts, and outcomes) through synthesis of evidence from published empirical studies (which may be qualitative, quantitative, or mixed methods research).

Not all initial programme theories developed from stage 1 and 2 will be ‘tested’ in stage 3 due to various issues like feasibility, obviousness, and lack of empirical evidence. Building the programme theories will require iterative discussions within the project team and with our stakeholders to make sense of and synthesise the different theories into initial coherent programme theories. Once the programme theories have been developed by the project team it will be presented to the stakeholders to obtain their feedback. We will refine the initial programme theories based on their feedback.

The prioritization of the theories (*especially between Stage 2 and Stage 3*) will be based on following criteria:

1. The perceived explanatory power of the programme theories, which means choosing theories which are thought to best explain the variation in the effectiveness and cost of admission avoidance intermediate care models.
2. Relevance and plausibility (to stakeholders)
3. Simplicity and clarity (including that they are logical)
4. Middle range (balance between the degree of abstraction/invisibility and specificity/concreteness)
5. Non-obvious (not too self-evident)
6. Inter linkage between theories

**Searches**

Searches for empirical studies to test the programme theories will be developed and conducted by an information specialist (SB) with extensive experience of conducting searches for complex systematic reviews, particularly realist reviews. We anticipate that we will search MEDLINE, MEDLINE In-Process, CINAHL, and the Health Management Information Consortium (HMIC) database. Additional bibliographic databases will be searched if deemed appropriate. Bibliographic database searches will combine both free-text terms (e.g., title and abstract) and controlled vocabulary (e.g., MeSH in MEDLINE). The specific search terms will depend on the content of the initial programme theory but is likely to include terms which describe MIPAC and similar models of care. We also anticipate checking the reference lists of relevant studies and conducting forward citation searching on key studies of interest.

**Type of studies to be included**

**Stage 1: Searching for programme theories**

The team (Sujan Rijal, Rob Anderson and Fan Yang) has considered several key details in formulation of inclusion criteria which include (not exhaustive): country/region/city, service setting (location of the units), types of services provided, patient groups, source of funding, organization of services (like availability of healthcare providers, scale of service, etc.), duration of the service provided, etc. Other details will be added, if deemed necessary. An inclusion criteria has been developed to screen the results from the database search. In the initial phase, the team will independently screen first 200 studies and consult to finalise the inclusion criteria before continuing with further screening (using Rayyan- a web-based screening tool for systematic reviews).

**Inclusion criteria**

| S.No | **Inclusion criteria** | **Comments** |
| --- | --- | --- |
| 1 | Studies published from 2000 onwards |  |
| 2 | Literature in English language or Norwegian | Except Norwegian |
| 3 | **Explanatory (in relation to outcome: effectiveness, improvement in care quality ) or ‘conceptually rich’ studies on hospital admission avoidance intermediate care (mostly qualitative) which** | Randomised control trials and other effectiveness studies (primarily concerned with measuring effectiveness and do not typically provide conceptually rich causal assertions) will be excluded |
| 3a | **Either are:**  Provides 24 hours inpatient acute care service for somatic patients 18 years or older  Managed at local or regional level  Lower level of care than specialised hospitals  High income countries with (dominantly) publicly funded health system | Intermediate care schemes which are not admission avoidance and are exclusively used as early supported discharge, step down care (***all or most patient referrals or transfers are from the community (people living in their own homes or care homes) and not from hospital are to be included)*** |
| 3b | **OR are:**  Municipal in-patient acute care (MIPAC) specific studies in Norway |  |
| 3c | **OR are:**  Previous realist reviews of intermediate care schemes |  |
| 4 | Policy documents, opinion pieces and commentaries on intermediate care schemes (MIPAC and that resemble MIPAC) | Papers like health acts, guidelines, etc. will be read for the purpose of familiarising with the rationale of the interventions/schemes and not for developing programme theories. |

***All the documents in Norwegian will be translated.***

**Stage 2: Developing programme theories**

Following types of documents/literatures will be used to identify and develop initial program theory (or theories):

- Previous realist reviews of hospital admission avoidance in-patient intermediate care models (or intermediate care models in general?).
- Conceptually rich, mostly qualitative, research studies that explicitly addresses or explains variations in the effectiveness of different admission avoidance intermediate care models or resource use in these models of care explaining why and how the models have (or not) been successful in achieving the intended outcomes.
- Government papers like status reports, policy documents, guidance materials, commentaries, opinion pieces, discussion, and individual works like evaluation reports, unpublished thesis work, etc.

**Stage 3: Refining programme theories**

For the refinement of programme theories, following types of studies will be included:

- Comparative quantitative evaluations (of outcome) of different models of admission avoidance intermediate care which report outcomes included from the beginning of the revie for development of programme theories.
- Comprehensive qualitative studies involving evaluations of process and implementation of models of care of interest.

**Participants/population**

The focus will be on (services/units for) any types of patient or people who would otherwise require an unplanned admission to a hospital as an inpatient (*see Intervention definition below*).

**Intervention(s)**

Any intermediate care model that has a primary goal of *providing 24-hour in-patient acute care to the people at the local/municipal level such that people who do not need specialised hospital inpatient services are treated at the local level*.

**Comparator(s)**

We are not necessarily comparing different models of admission avoidance intermediate care to assess relative effectiveness. Comparison will mainly be between similar programme theories in different setting/contexts or between rival programme theories with same empirical evidence. For Stage 3, however, the actual or implied comparator will be the inpatient admissions to acute hospitals that have been avoided as a consequence of having an intermediate care inpatient unit at a local level.

**Context**

The primary context of the review is the shift from use of specialized hospital-based health care services to health care service funded at the local/primary level, as much as possible. This is believed to reduce the burden on specialised health care service delivery by avoiding unnecessary hospitalizations, potentially resulting in realisation of a more sustainable health care delivery system.

**Main outcome(s) of interest**

The effectiveness and costs (use of resources) in different models of hospital admission avoidance in-patient intermediate care. The specific types of outcomes or effectiveness measure will emerge from the programme theories. However, if we have to be selective, we will aim to focus on outcomes that are more valued by and of known importance to patients and their carers (as opposed to these perceived to be important by clinicians or services). This may mean (for example) that we prioritise inclusion of studies that use patient-reported or carer-reported outcome measures, over studies that only report clinician-assessed care improvements.

**Additional outcome**

None

**Data extraction (selection and coding)**

**Stage 1: Searching for programme theories**

There is no standardised data extraction in stage 1 except the exploration, collection, and organisation of potential programme theories.

**Stage 2: Developing programme theories**

Data extraction will be done for two different purposes. Firstly, data extraction to develop programme theories will be done by inspecting documents from stage 1 for explanations or causal statements about the effectiveness of admission avoidance intermediate care models and costs of such models. This will primarily be qualitative in nature and will be extracted precisely before coding them to develop initial list of program theories. An inductive approach will be used in coding and listing the initial program theories.

**Stage 3: Refining programme theories**

A second stage of data extraction in this review will seek to extract data on specific components of similar or rival programme theories (i.e., Stage 3). This will focus on those elements which are either directly observable like certain outcomes (e.g., effectiveness) or indirectly implied through the information in the study. This includes capturing information in the form of statement or paragraphs making assertions about outcomes or underlying mechanisms of one or more programme theories.

Data extraction will be done primarily using MS-Excel.

**Study quality assessment**

For studies used to develop initial programme theories, assessment will be done on the basis of conceptual richness. Any papers or studies which informs stage 2 will not be formally assessed methodologically as their value lies in the clarity and coherence of the theoretical and conceptual insights offered, and the extent to which these build on the emerging programme theories.

For the final stage in the review, primary research studies will be selected for empirical evidence. These studies will be assessed using different tools based on the type of studies. Studies dealing with comparative quantitative outcome evaluations will be assessed by EPHPP tool from Effective Public Healthcare Panacea Project . Similarly, the Consensus Health Economic Criteria (CHEC) list will be used to assess the quality of economic evaluation studies. For studies containing statistical analysis of non-randomised single cohort studies/cross sectional studies , modified Newcastle Ottawa Scale (NOS) will be used. Lastly, qualitative research (also mixed method) will be assessed using the Wallace criteria. This has been illustrated in the table below.

| **Type of empirical study** | **Name of QA tool** | **Link to current version & and key citation:** |
| --- | --- | --- |
| Comparative quantitative outcome evaluations | Effective Public Healthcare Panacea Project (**EPHPP** tool) | <https://www.ephpp.ca/quality-assessment-tool-for-quantitative-studies/>  Thomas, B.H., Ciliska, D., Dobbins, M., & Micucci, S. A process for systematically reviewing the literature:  Providing the research evidence for public health nursing interventions. *Worldviews on Evid Based Nurs*. 2004; 1(3):176-184. |
| Economic evaluations | **CHEC**  list (Consensus Health Economic Criteria) | <https://www.maastrichtuniversity.nl/research/caphri/our-research/creating-value-based-health-care/chec-list-consensus-health-economic>  Evers S, Goossens M, de Vet H, van Tulder M, Ament A. Criteria list for assessment of methodological quality of economic evaluations: Consensus on Health Economic Criteria. *Int J Technol Assess Health Care*. 2005 Spring;21(2):240-5. PMID: 15921065. |
| Non-comparative evaluations (e.g. regression analysis or statistical analysis of non-randomised single cohort studies/cross sectional studies) | Modified Newcastle-Ottawa Scale (NOS) | <https://www.ohri.ca/programs/clinical_epidemiology/oxford.asp> |
| Qualitative research studies and evaluations  (also OK for mixed methods studies) | The ‘Wallace criteria’ | <https://bristoluniversitypressdigital.com/view/journals/pp/32/4/article-p455.xml>  Wallace, A., Baldwin, S., Croucher, K., & Quilgars, D. (2004). Meeting the challenge: developing systematic reviewing in social policy, Policy & Politics, 32(4), 455-470. |

**Synthesis of evidence**

**Stage 1: Searching for programme theories**

There is no formal evidence synthesis involved in this stage of the review.

**Stage 2: Developing programme theories**

Synthesis of evidence within a realist review is an iterative and dominantly interpretative process. This will be done in two different phases of the realist review. First, synthesis will involve the reviewer along with the review team going through the studies and grouping the statements from sources that imply causal assertions or explanations. As mentioned, this is an iterative process and will involve multiple discussions within the review team and through sense-checking with stakeholders (healthcare providers in MIPAC and evaluators when and where feasible and appropriate). A refined version of list of programme theories that are clear, simple, plausible, and logical will be finalized.

Ultimately, by Stage 2 of the review, the program theories will ideally be expressed in terms of underlying Mechanisms, relevant Contexts and Outcomes (related Ms, Os, and the Cs that enable/hinder the generation of Os from Ms).

After the completion of this stage, the most relevant programme theories will be selected to the focus for testing against empirical evidence in the next stage of the review.

**Stage 3: Refining programme theories**

The final stage of evidence synthesis (although the process is not linear and can go through several iterations), is done using evidence from primary research (either qualitative or quantitative) to refine or “test” the programme theories and are ideally expressed as Context-Mechanism-Outcome configurations. Data from relevant studies will be extracted in the form of tabulations and annotations. The data from these extractions will be used to:

- Question the integrity of the program theory (validation of the causal assertions and explanation)
- Compare same program theory in different contexts/settings
- Analyse rival program theories and make judgement
- Compare initial aim/goal of the policy implementation, expectation (how it was envisioned to work) with the actual practice information, and experiences of those who are involved in delivery of the care.

At the end of this stage, several refined program theories will be developed with detailed explanation of how different combinations of mechanisms and underlying contexts result in the outcomes revealed in the empirical studies.

**Stakeholder involvement and PPI**

The review will make use of knowledge and experiences of various personnel involved from the initiation, delivery, implementation to evaluation of admission avoidance in-patient intermediate care models in Norway. They will play a supporting role in the realist review from development of initial program theories to refining them. The stakeholders include:

- Health care providers in intermediate care services like physicians (both from hospitals and primary care) and nurses.
- Health actors like policy makers, officials from municipalities, and people from allocation offices.
- Health service research specialists from the University of Oslo, University of Exeter, Akershus University Hospital who have recognised experience in health research and intermediate care.

We also hope that the stakeholders will also provide ‘internal’ peer review of the final report of the realist review which will be circulated by the review team.

**Publication and dissemination**

Dissemination of the realist review will be done with the aim of informing health policy makers and service managers in Norway and other high-income countries with publicly funded health systems, by providing them information and knowledge about how and in what contexts the current model of admission avoidance in-patient intermediate care model works. This will include refined programme theory about the costs of providing and the cost-related consequences of the outcomes of inpatient hospital admission avoidance programmes. The findings of the review can be used to discuss current practice and challenges in successful implementation of the care model. Similarly, they can be used to suggest possible ways of improving the model, for example by identifying which core underlying mechanisms are most likely associated with intended outcomes in different circumstances. Lastly, the findings can be used to devise new policy and models of admission avoidance in-patient intermediate care.

As far as dissemination is concerned, it will be done through following channels:

- A publication in peer-reviewed journal (planned- Journal of Health Services Research and Policy)
- A comprehensive research report that will include detailed description of methods and results from the review

**References**

ANDERSON, R. 2008. New MRC guidance on evaluating complex interventions. British Medical Journal Publishing Group.

BERG, R. & NANAVATI, J. 2016. Realist review: current practice and future prospects.

COLEMAN, E. A. & BOULT, C. 2003. Improving the quality of transitional care for persons with complex care needs. *J Am Geriatr Soc,* 51**,** 556-7.

HOOT, N. R. & ARONSKY, D. 2008. Systematic Review of Emergency Department Crowding: Causes, Effects, and Solutions. *Annals of Emergency Medicine,* 52**,** 126-136.e1.

JOHANNESSEN, A. K. & STEIHAUG, S. 2020. The function of the Norwegian municipal acute units fails to fulfill the intention of health authorities. *Scand J Prim Health Care,* 38**,** 75-82.

NYSTRØM, V., LURÅS, H., MIDLÖV, P. & LEONARDSEN, A.-C. L. 2021. What if something happens tonight? A qualitative study of primary care physicians’ perspectives on an alternative to hospital admittance. *BMC Health Services Research,* 21**,** 447.

NYSTRØM, V., LURÅS, H., MOGER, T. & LEONARDSEN, A.-C. L. 2022. Finding good alternatives to hospitalisation: a data register study in five municipal acute wards in Norway. *BMC Health Services Research,* 22**,** 715.

PAWSON, R., GREENHALGH, T., HARVEY, G. & WALSHE, K. 2005. Realist review--a new method of systematic review designed for complex policy interventions. *J Health Serv Res Policy,* 10 Suppl 1**,** 21-34.

PEARSON, M., HUNT, H., COOPER, C., SHEPPERD, S., PAWSON, R. & ANDERSON, R. 2013. Intermediate care: a realist review and conceptual framework. *Final report. NIHR Service Delivery and Organisation Programme*.

PEARSON, M., HUNT, H., COOPER, C., SHEPPERD, S., PAWSON, R. & ANDERSON, R. 2015. Providing effective and preferred care closer to home: a realist review of intermediate care. *Health & social care in the community,* 23**,** 577-593.

RCN. 2021. *Variations in trajectories, utilisation, and costs in Norwegian municipal in-patient acute care units* [Online]. Available: <https://prosjektbanken.forskningsradet.no/en/project/FORISS/324959?Kilde=FORISS&distribution=Ar&chart=bar&calcType=funding&Sprak=no&sortBy=date&sortOrder=desc&resultCount=30&offset=0&TemaEmne.2=Forskning+for+fornyelse+av+offentlig+sektor> [Accessed].

SEZGIN, D., O’CAOIMH, R., O’DONOVAN, M. R., SALEM, M. A. & KENNELLY, S. E. A. 2020. Defining the characteristics of intermediate care models including transitional care: an international Delphi study. *Aging Clinical and Experimental Research,* 32**,** 2399-2410.

SKINNER, M. S. 2015. Døgnåpne kommunale akuttenheter: En helsetjeneste-modell med rom for lokale organisasjonstilpasninger - Municipal acute bed units: a national health service model with scope for local adaptation. *Tidsskrift for omsorgsforskning,* 1**,** 131-144.

STEINER, A. 2001. Intermediate care—a good thing? *Age and Ageing,* 30**,** 33-39.

SWANSON, J. O. & HAGEN, T. P. 2016. Reinventing the community hospital: a retrospective population-based cohort study of a natural experiment using register data. *BMJ Open,* 6**,** e012892.

VATNØY, T. K., SKINNER, M. S., KARLSEN, T.-I. & DALE, B. 2020. Correction to: Nursing competence in municipal in-patient acute care in Norway: a cross-sectional study. *BMC Nursing,* 19**,** 122.

WHO 2016. Transitions of care.

YOUNG, J. 2009. The development of intermediate care services in England. *Archives of Gerontology and Geriatrics,* 49**,** S21-S25.
